# On the Importance of Individualized, Non-Coplanar Beam Configurations in Mediastinal Lymphoma Radiotherapy, Optimized With Automated Planning

**DOI:** 10.3389/fonc.2021.619929

**Published:** 2021-04-15

**Authors:** Linda Rossi, Patricia Cambraia Lopes, Joana Marques Leitão, Cecile Janus, Marjan van de Pol, Sebastiaan Breedveld, Joan Penninkhof, Ben J.M. Heijmen

**Affiliations:** Department of Radiotherapy, Erasmus MC Cancer Institute, Rotterdam, Netherlands

**Keywords:** automated multi-criterial planning (MCO), comparison, non-coplanar angle, VMAT versus IMRT, number of beams, mediastinal lymphoma, individualized beam angle optimization, personalized radiotherapy

## Abstract

**Background and Purpose:**

Literature is non-conclusive regarding selection of beam configurations in radiotherapy for mediastinal lymphoma (ML) radiotherapy, and published studies are based on manual planning with its inherent limitations. In this study, coplanar and non-coplanar beam configurations were systematically compared, using a large number of automatically generated plans.

**Material and Methods:**

An autoplanning workflow, including beam configuration optimization, was configured for young female ML patients. For each of 25 patients, 24 plans with different beam configurations were generated with autoplanning: 11 coplanar CP_x plans and 11 non-coplanar NCP_x plans with x = 5 to 15 IMRT beams with computer-optimized, patient-specific configurations, and the coplanar VMAT and non-coplanar Butterfly VMAT (B-VMAT) beam angle class solutions (600 plans in total).

**Results:**

Autoplans compared favorably with manually generated, clinically delivered plans, ensuring that beam configuration comparisons were performed with high quality plans. There was no beam configuration approach that was best for all patients and all plan parameters. Overall there was a clear tendency towards higher plan quality with non-coplanar configurations (NCP_x≥12 and B-VMAT). NCP_x≥12 produced highly conformal plans with on average reduced high doses in lungs and patient and also a reduced heart Dmean, while B-VMAT resulted in reduced low-dose spread in lungs and left breast.

**Conclusions:**

Non-coplanar beam configurations were favorable for young female mediastinal lymphoma patients, with patient-specific and plan-parameter-dependent dosimetric advantages of NCP_x≥12 and B-VMAT. Individualization of beam configuration approach, considering also the faster delivery of B-VMAT vs. NCP_x≥12, can importantly improve the treatments.

## Introduction

Patients treated with a combination of multi-agent chemotherapy and radiation for Hodgkin or non-Hodgkin lymphoma are mostly young at diagnosis. About 80% of these patients achieve long-term remission. Given the age at diagnosis and the favorable long-term prognosis, therapy-related late effects including secondary malignancies (1–7) and cardiovascular disease (8–12) have become increasingly important. In recent years, radiotherapy (RT) for lymphoma has evolved by considerably decreasing target volumes (from extended field to involved field to involved site or involved node) and radiation doses (from 40 to 30 Gy or even 20 Gy in selected cases). These factors contribute to a decrease in the risk of late toxicity (1, 6, 13–16).

Applied radiotherapy techniques have also evolved, with intensity modulated radiation therapy (IMRT) and volumetric modulated arc therapy (VMAT) emerging as alternatives to 3D conformal RT (3D-CRT). In this context, the typical low-dose bath of VMAT plans has been pointed at as a cause of concern, as it could increase the risk of secondary cancers relative to 3D-CRT (17). The low-dose bath in the lungs has also been associated with increased risk of radiation pneumonitis (18). Choice of beam arrangement may impact plan quality. This has been investigated in detail for ‘butterfly’ beam arrangements that can contain non-coplanar beams. In particular, the (non-coplanar) B-VMAT approach described by Fiandra et al. (19) has shown to reduce breast Dmean and V4Gy compared to VMAT, leading to similar calculated lower risks of secondary breast cancer as 3D-CRT (but risk of lung cancer relatively higher), as well as a lower risk of cardiac toxicity, in a group of patients with largely non-bulky disease, without axillary involvement (20). Voong et al. (21) observed a reduction in heart dose (but not in breast dose) by using five to seven IMRT beams (butterfly) with eventually one non-coplanar beam, relative to 3D-CRT in patients without bilateral axillary involvement. Proton therapy has also been proposed for further reductions of late toxicity in selected lymphoma patients (17, 22–25).

Current literature is non-conclusive regarding the optimal choice of RT treatment technique. The International Lymphoma Radiation Oncology Group (26) has benchmarked the best practice of 10 centers in 2013, showing that (i) the applied (photon) RT technique varied largely between institutions leading to large differences in the low-dose volumes, and (ii) in practice, difficult cases were often not planned according to the standard. The authors could not provide universal/consensus recommendations. Moreover, different authors pointed at the necessity for individualized selection of planning technique (19, 21, 27). This was in part attributed to the high heterogeneity in tumor location, shape, and size, as well as patient characteristics.

It is well known that manually generated treatment plans may suffer from inter-and intra-planner quality variations (28, 29). Moreover, finding optimal beam configurations with trial-and-error planning is extremely complex and time-consuming. On the other hand, the large anatomical variability in lymphoma patients (target size/shape and position) is a real challenge for development of a system for automated planning, where the aim is to generate a unique workflow that works well for all patients without further interactive fine-tuning of plans by a user. The issues with manual beam angle selection put heavy constraints on the number of beam configurations that were compared in published ML planning studies, and on the total number of included plans. To the best of our knowledge, in all published studies comparing beam configurations for treatment of lymphoma patients, beam angle class solutions (e.g., B-VMAT) were investigated, or beam angles were selected by planners, i.e. there was no patient-specific computer optimization of angles. So far, only the study by Clemente et al. (30) reported on autoplanning for lymphoma patients, but this did not include optimization of beam directions. Moreover, their workflow worked for OAR sparing, while there were limitations for PTV doses.

In this work, we used a large number of automatically generated plans for comparison of radiotherapy beam configurations for young females with ML. To this purpose, an automatic workflow for IMRT/VMAT plan generation, including integrated coplanar or non-coplanar beam angle and beam profile optimization for IMRT, was implemented and validated. The system was used to systematically compare plan quality differences between 24 coplanar and non-coplanar beam configuration approaches for 25 study patients.

## Materials and Methods

### Patients and Clinical Protocol

The study was based on a database with contoured planning CT-scans and manually generated, clinically delivered plans (CLIN) of 26 previously treated female ML patients (21 Hodgkin lymphoma and 4 B cell non-Hodgkin lymphoma). As explained in detail below, one patient (patient 0) was excluded from population-based analyses, leaving 25 evaluable patients for such analyses (patients 1–25).

Visual inspection of planning CT-scans ensured a heterogeneous selection of anatomical presentations in the patient cohort (superior/inferior mediastinum, with/without involvement of supraclavicular or axillar nodes, bulky disease, complex anatomy; see **Figure B1 in Electronic Supplement B**). The median patient age was 27 (range, 19–50). The PTV volumes varied from 97 to 1654 cc (median 605 cc). The prescription dose was 30 Gy in 15 fractions, excluding the sequential boost applied for some patients (3 × 2 Gy), which was not considered in this study.

In clinical practice, dosimetric aims were largely based on published recommendations (11, 12, 18, 31, 32). At least 95% of the target (ideally 100%) had to be covered by 95% of the prescribed dose (V95% >95%), while respecting the PTV over- and under-dose criteria; V110% <1% and V<90% <5 cc (preferably <2 cc), respectively. OAR requirements were the following, where a preferred value is indicated in parentheses: breast Dmean <5 Gy (<2 Gy), heart Dmean <26 Gy (<10 Gy), lungs Dmean <15 Gy (<13.5 Gy), lungs V5Gy <55% (<50%), and lungs V20Gy <30%. None of the planning requirements was truly a hard constraint (except for PTV V95%), i.e., depending on patient anatomy, violations were sometimes accepted. The 60% isodose was clinically evaluated (visually, not quantitatively), especially related to dose in the back/neck muscles. Five patients were treated with a coplanar partial-arc VMAT plan, and 20 patients were treated with a coplanar IMRT plan with, on average, 6.0 manually selected mediastinal beams (range, 4–8), mainly from (or close to) anterior and posterior directions (butterfly). For patients with neck involvement, one to four beams from (close to) lateral directions were added for neck irradiation only.

### Automated Plan Generation

An automated planning workflow for young ML patients was developed following the clinical planning aims described above. The core of the system was Erasmus-iCycle, an in-house developed multi-criteria optimizer featuring integrated beam angle and profile optimization (33), coupled to a Monte Carlo dose calculation engine (34). Pareto-optimal plans with clinically favorable trade-offs between all treatment requirements were realized with the optimization protocol [‘wish-list’ (33),] reported and explained in **Electronic Supplement B**. All plans for all patients were automatically generated with the same wish-list without any manual fine-tuning.

For coplanar beam angle optimization (BAO), the candidate beam set consisted of 36 equiangular beams (0°, 10°, …, 350°). For non-coplanar BAO, beam candidates, defined by all combinations of beams with 10 degree separation from each other in all directions, were verified at the linac to exclude beams with (potential) collisions between the patient/couch and the gantry, ending up with a set of 194 candidate beam directions (including the 36 coplanar beams). The applied beam energy was 6 MV.

#### Compared Beam Configurations

**Figure 1:**
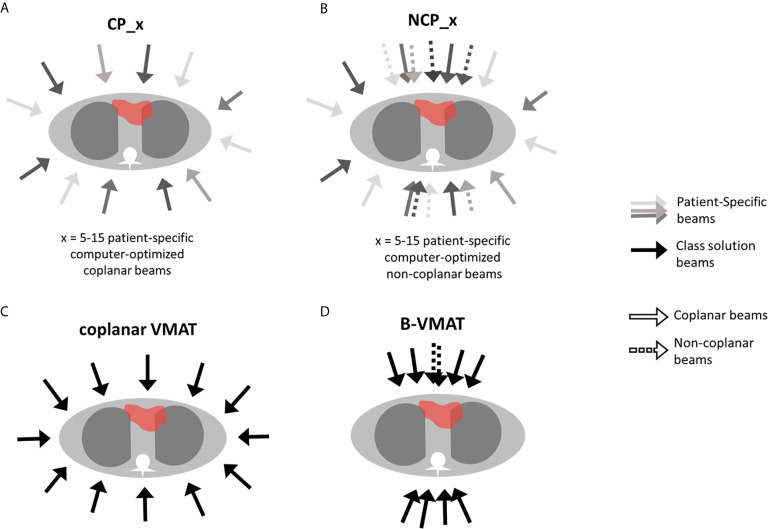
Schematic representation of the investigated beam configurations: CP, coplanar **(A)**, NCP, non-coplanar **(B)**, coplanar VMAT **(C),** and B-VMAT, Butterfly-VMAT **(D)**.

For all 25 study patients, the following 24 autoplans were generated to systematically investigate the impact of beam angle configuration on plan quality (see also **Figure 1**):

- CP_x: coplanar plans with x = 5–15 beams with computer-optimized, patient-specific directions.
- NCP_x: non-coplanar plans with x = 5–15 beams with computer-optimized, patient-specific directions.
- VMAT: IMRT plan with 21 coplanar equiangular beams, reproducing full-arc VMAT dose distribution (35).
- B-VMAT: non-coplanar class solution, consisting of 20 IMRT beams equally spread in three 60° arcs, two centered at gantry angle = 0° and 180°, with couch 0° (with seven beams each arc, with 10° separation space) and one centered at gantry angle = 0° with couch = 90° (with six beams, with 10° separation space, excluding angle gantry 0° and couch 90°, already present in the anterior arc), mimicking the butterfly geometry described by Fiandra et al. (19).

#### Plan Evaluations and Comparisons

Plans were mainly evaluated and compared using PTV and OAR planning goals applied in clinical planning (above). On top of that we also reported on breast(s) V4Gy (19), PTV V107%, conformity index (CI, defined as patient V95%/PTV volume), and patient V5Gy (cc) and V20Gy (cc), where the patient is defined by the external skin structure. PTV V110%, mentioned in the clinical planning protocol, was always far below the requested 1%, and was therefore not reported. Two-sided Wilcoxon signed-rank tests were used for statistical analyses, with p-values lower than 0.05 indicating statistical significance in plan parameter differences.

## Results

### Quality of Autoplans

Prior to the comparisons of beam angle configurations, several analyses were performed to ensure that the autoplans used for these comparisons were clinically acceptable and of high quality. Data is partly presented below, and partly in **Electronic Supplement A**.

From the 624 autoplans defined in the M&M section (24 plans for all 26 patients), 617 (98.9%) satisfied the clinical PTV coverage requirement, i.e. V95% ≥ 95%. The seven autoplans with insufficient PTV coverage were from the same patient (patient 0 in **Figure B1 in Electronic Supplement B**), all with relatively low numbers of coplanar beams (CP_5-11). In the IMRT plan used for treatment of this patient, sufficient PTV coverage was obtained at the cost of exceptionally high breast and heart doses: breast Dmean = 11.9/6.3 Gy left/right, and heart Dmean = 23.2 Gy (by far the highest in the group), all strongly exceeding clinical thresholds. The wish-list for autoplanning (**Table B1 in Electronic Supplement B**) was developed to balance OAR vs. PTV dose, which could result in too low PTV coverage to protect OARs. For 25/26 patients, all autoplans had sufficient coverage while also avoiding constraint violations. As indicated above, for patient 0, 17/24 plans had adequate coverage, the remaining seven had not. To avoid patient group analyses with unacceptable plans, patient 0 was not in such analyses in the remainder of the paper and the Electronic supplements, leaving 600 evaluable plans. The relevance of the proposed autoplanning workflow for patient 0 is further discussed in the Discussion section.

Automatically generated plans had overall favorable plan parameters compared to clinically delivered plans, generated with manual planning (**Table 1**, further analyses in **Electronic Supplement A, section A1**). **Table 1** compares mean autoplan parameters with the corresponding mean parameters for the CLIN plans. All averaged PTV dose parameters of the autoplans were favorable compared to those of the CLIN plans. The values for mean/minimum PTV coverage went up from 98.1%/95.0% to 99.5%/97.1%. A remarkable reduction in PTV V<90% was observed, with mean/maximum values decreasing from 2.9 cc/19.0 cc to 0.5 cc/6.6 cc. Autoplans were also superior to CLIN in all mean OAR plan parameters. For lungs and patient, observed maximum values in the autoplans were slightly higher than those in the CLIN plans. This could be related to the improved PTV dose, but statistics might also contribute here: the more plans generated, the higher the chance on outliers (25 CLIN plans vs. 600 autoplans).

**Table 1:** Comparisons of mean (and ranges) autoplan parameters (units are given in parameter column) with corresponding mean (and ranges) clinically delivered plan (CLIN) parameters for patients 1–25, and absolute differences (mean and ranges).

Structure	Parameter	25 CLIN plans		600 Autoplans		Abs. differences (CLIN-auto)	
		Mean	Range	Mean	Range	Mean	Range
PTV	V95% (%)	**98.1**	95.0–99.7	**99.5**	97.1–99.9	−**1.3**	−4.8 to 0.5
	V<90% (cc)	**2.9**	0.0–19.0	**0.5**	0.0–6.6	**2.4**	−2.1 to 18.8
	V107% (%)	**0.9**	0.0–2.6	**0.2**	0.0–5.4	**0.7**	−4.5 to 2.6
	CI	**1.2**	1.1–1.6	**1.2**	1.1–1.5	**0.0**	−0.2 to 0.2
Breast_R_	Dmean (Gy)	**1.9**	0.1–6.2	**1.6**	0.2–5.2	**0.3**	−1.6 to 3.4
	V4Gy (%)	**10.7**	0.0–35.5	**7.4**	0.0–33.6	**3.2**	−15.6 to 24.8
Breast_L_	Dmean (Gy)	**1.9**	0.0–5.4	**1.6**	0.1–5.1	**0.2**	−1.9 to 3.6
	V4Gy (%)	**10.0**	0.0–37.7	**8.4**	0.0–38.2	**1.6**	−12.3 to 31.4
Heart	Dmean (Gy)	**6.5**	0.2–20.0	**5.6**	0.2–20.4	**0.9**	−3.3 to 5.9
Lungs	Dmean (Gy)	**8.3**	2.1–14.3	**7.4**	1.8–16.8	**0.9**	−2.4 to 3.3
	V5Gy (%)	**44.2**	10.6–79.5	**38.9**	7.4–88.2	**5.3**	−15.6 to 25.1
	V20Gy (%)	**15.7**	3.0–30.4	**14.4**	2.6–39.2	**1.3**	−11.5 to 8.9
Patient	V5Gy (cc)	**5135.6**	1191.8–	**4861.3**	960.5–10816.9	**273.6**	−978.0 to 2238.7
	V20Gy (cc)	**1869.8**	271.1–10186.8	**1809.6**	259.5–5056.6	**59.6**	−1128.9 to 1145.6

For calculation of the mean autoplan parameters, all 24 plans per patient, used for the beam configuration studies, were considered. Mean autoplan parameters compared favorably with mean CLIN parameters.

As discussed in Electronic appendix A, section A2, involved clinicians rated positively the automatically generated plans.

### Comparisons of Beam Configurations

All analyzed 600 autoplans for patients 1 to 25 showed highly comparable PTV doses (standard deviations for V95%, V<90%, and V107% were 0.2%, 0.4 cc, and 0.3%). Therefore, only OAR doses are reported in this section.

**Figure 2:**
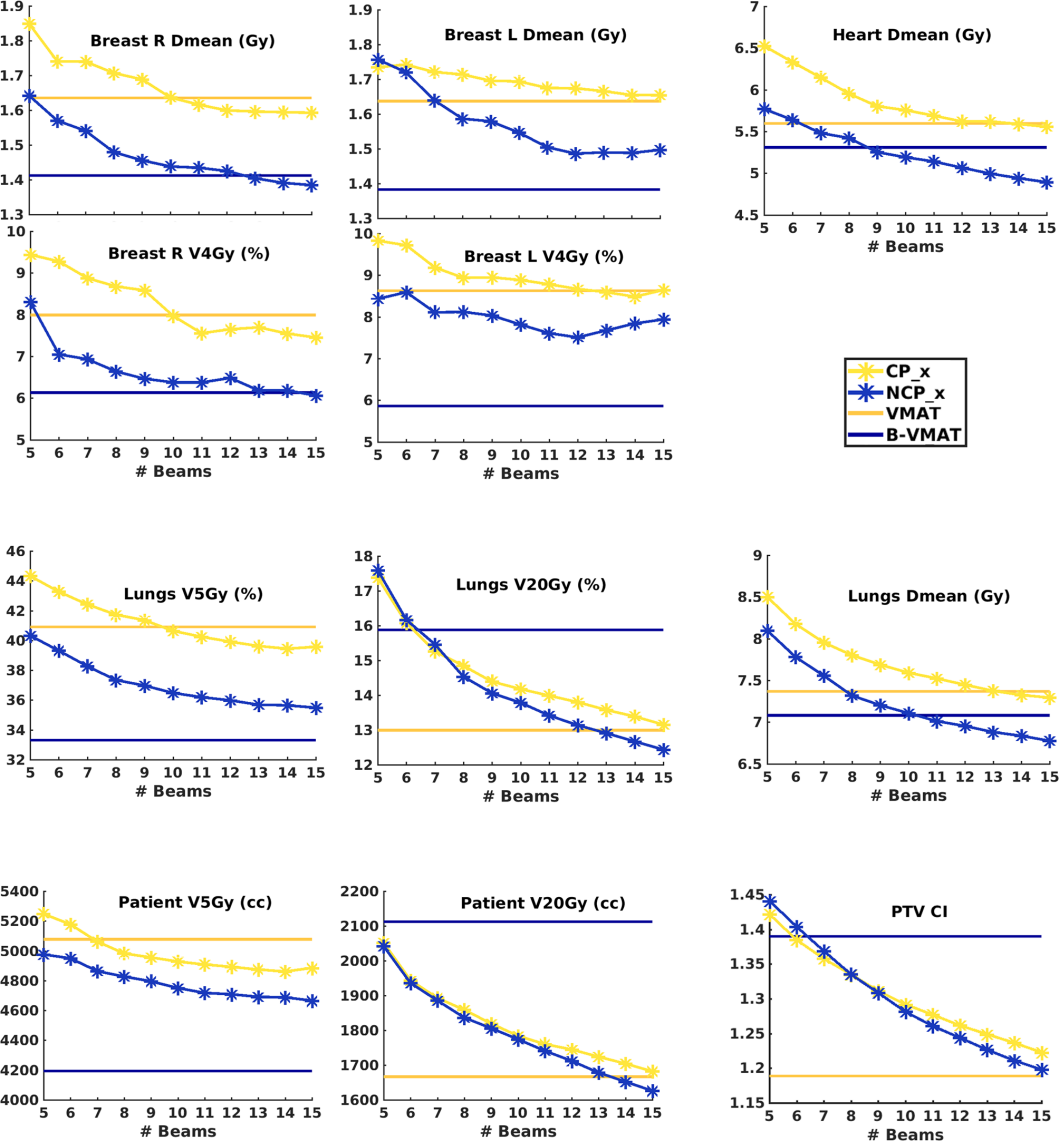
Population mean dosimetric plan parameters for CP_x and NCP_x as a function of the number of beams per plan (x). The dashed horizontal lines indicate the population mean values for VMAT and B-VMAT. p-Values for beam configuration comparisons are presented in **Figure B2 in Electronic Supplement B**.

**Figure 3:**
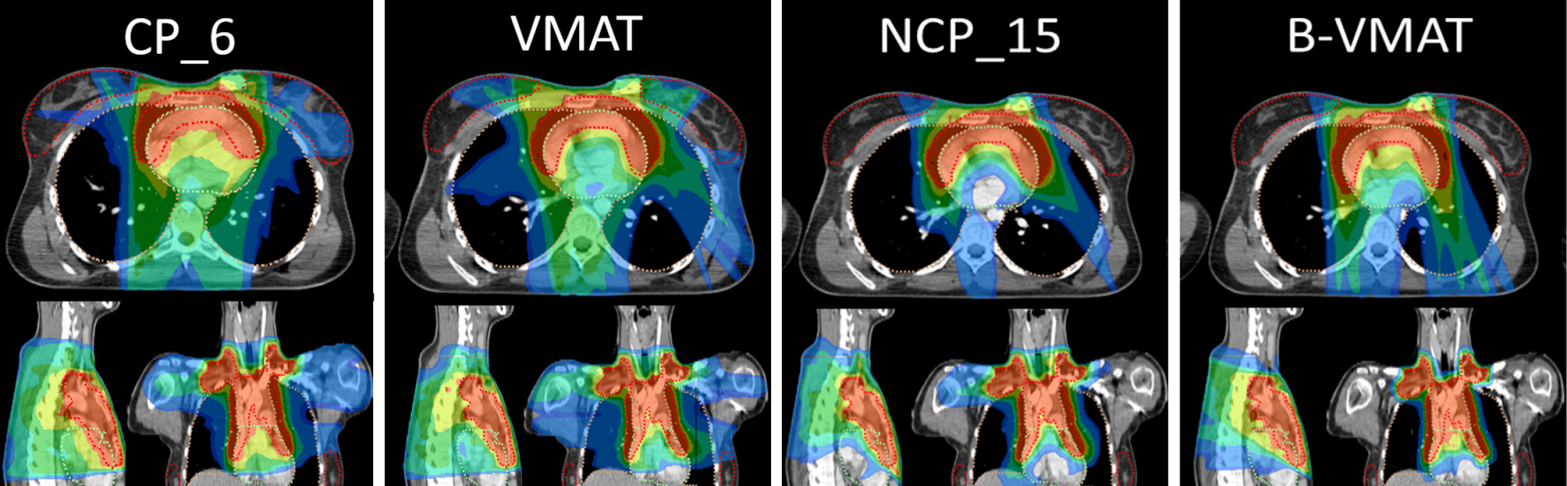
Dose distributions for patient 3. CP_6 was added as, on average, six beams were used clinically. CP_15 was similar to VMAT and was therefore not added. The isodose lines are percentages relative to the prescribe dose, i.e., 100% = 30 Gy, with color legend as light blue, 16.7% (5 Gy as OAR constraints); azure, 20%; light green, 40%; dark green, 60%; yellow, 80%; red, 95%.

**Figure 4:**
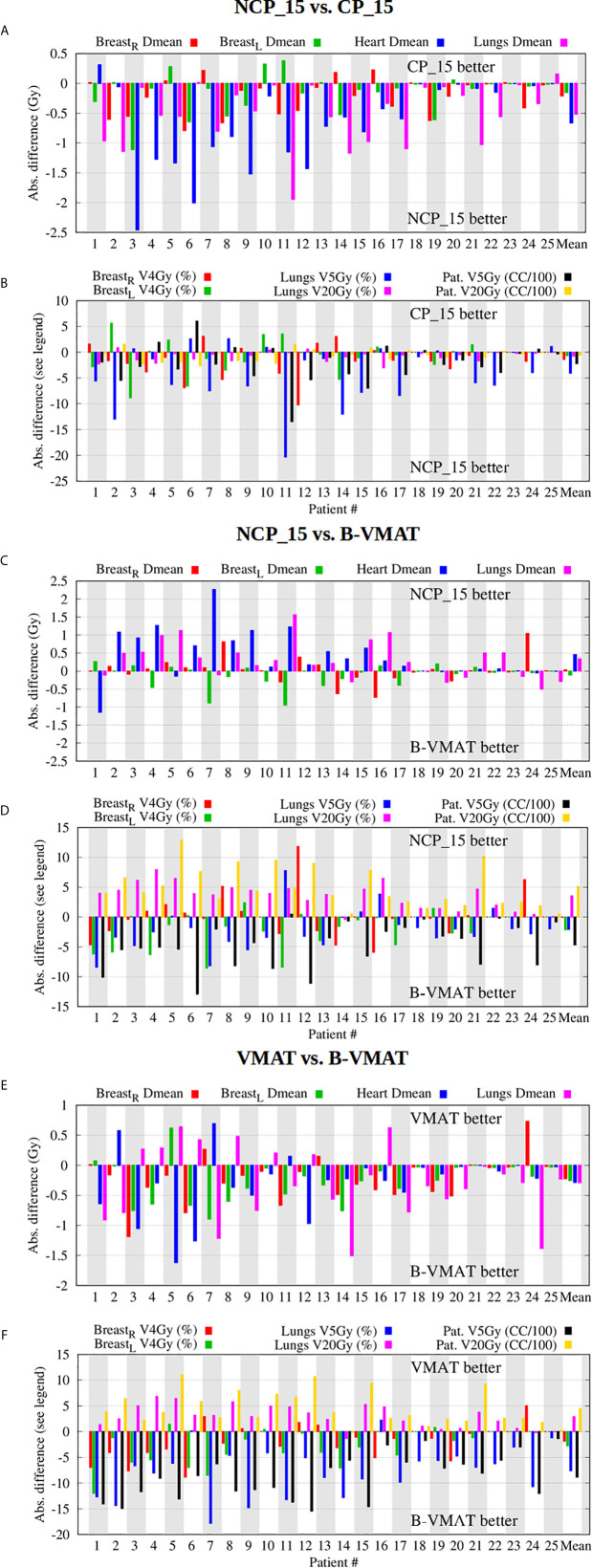
Beam configuration comparisons - NCP_15 vs. CP_15 **(A, B)**, NCP_15 vs. B-VMAT **(C, D)**, and VMAT vs. B-VMAT **(E, F)** - showing large inter-patient and inter-parameter variations in differences in plan parameter values. Patients were ordered according to descending heart Dmean in the NCP_15 plans.

**Figure 5:**
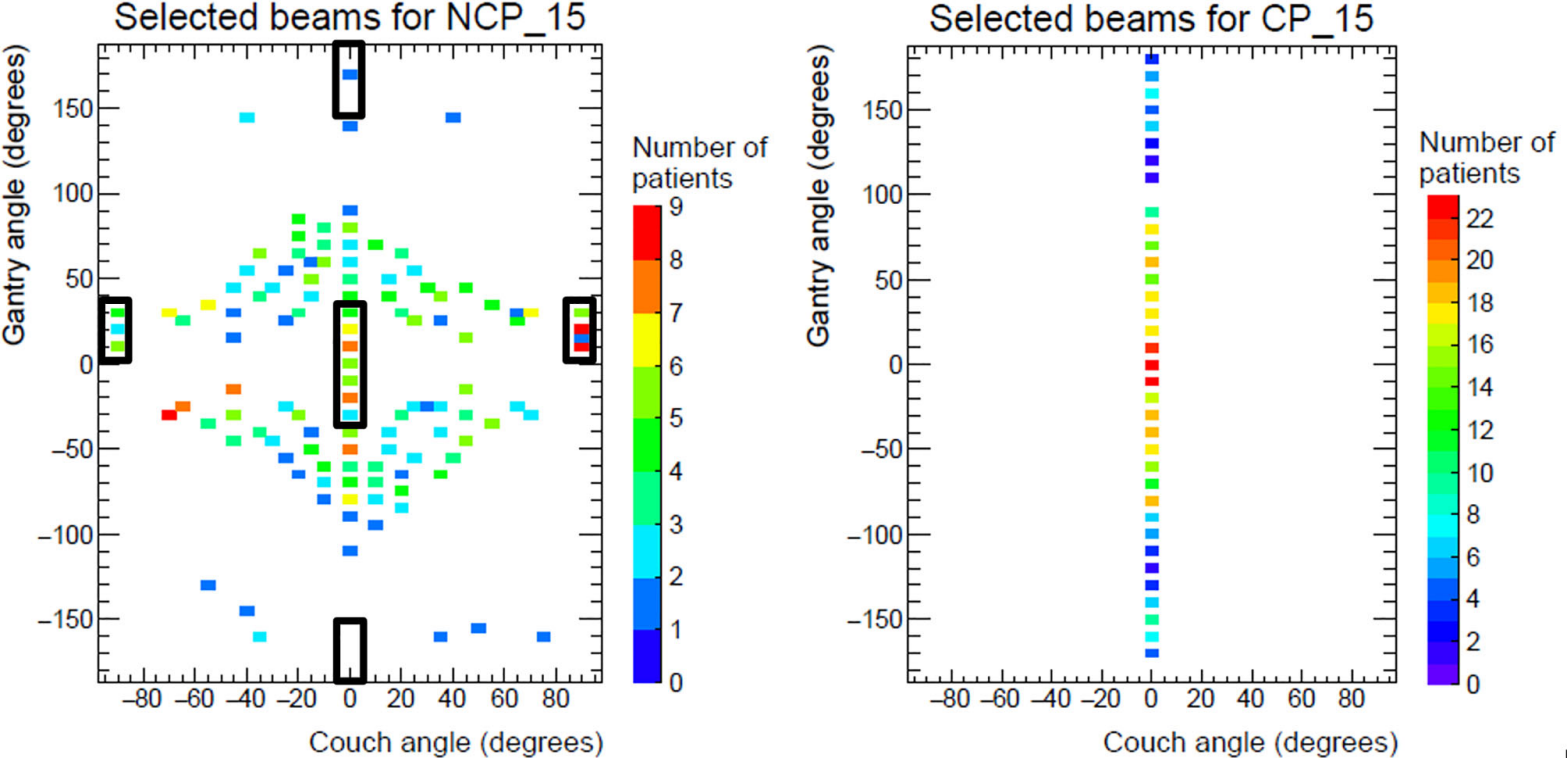
Population distributions of beams selected for NCP_15 (left) and CP_15 (right) for patients 1 to 25 (375 beams per panel). The black rectangles in the left panel indicate the beams present in B-VMAT. Note: B-VMAT beams in the rectangular at couch -90 degrees are in reality delivered with couch angle +90 degrees, while flipping the respective gantry angles to negative values.

**Figure 2** shows population average plan parameters for VMAT, B-VMAT and CP_x and NCP_x (x = 5–15) (p-values for all mutual comparisons are reported in **Figure B2 in Electronic Supplement B**). Below, the main observations are summarized:

- *Beam number x in NCP_x and CP_x*: Both for CP_x and NCP_x plan quality increased with increasing x. For some parameters there was some leveling off for x≥11 beams, but not for all. Improvements obtained by adding a beam were highly statistically significant for high-dose plan parameters, i.e. lungs and patient V20Gy, heart Dmean and lungs Dmean. For medium dose parameters (lung V5Gy and breast Dmean) differences were almost always statistically significant. Improvements in left breast V4Gy were not statistically significant.
- *NCP_x vs. CP_x*: For equal beam numbers, x, NCP was always better than CP. **Figures 4A**, **B** show that plan improvements with NCP_15 compared to CP_15 were observed for all patients, although the gain was clearly patient and plan parameter dependent. Differences in mean values were often considered clinically significant.
- *NCP_x vs. VMAT*: NCP_x≥10 was better than or equal to VMAT for all OAR plan parameters. For many parameters, equality was achieved for much less beams.
- *NCP_x vs. B-VMAT*: NCP_x was overall superior for lungs and patient V20Gy and for conformality (CI) (higher doses), and, for larger x, also for heart Dmean and lungs Dmean. **Figures 4C**, **D** show that differences are strongly patient- and parameter dependent. Possibly patients that may benefit most from NCP over B-VMAT in terms of heart or lungs doses are those with targets extending to the lower mediastinum (e.g., pt. 4, **Figure B1**) and/or the supraclavicular region bilaterally (pts. 3,5,11), or with asymmetrical target relative to the midline (e.g., unilateral axilla, pt. 16). Overall, B-VMAT had lower left breast Dmean and V4Gy, lungs V5Gy and patient V5Gy (lower dose parameters). However, some patients did benefit from the individualized beam choice in terms of breast dose, such as patients with axillar involvement (e.g., pts. 8 and 24) and with asymmetrical targets relative to the midline (e.g., pt. 12).
- *VMAT vs. B-VMAT*: Lungs V20Gy, patient V20Gy and CI (higher dose parameters) were on average lowest with VMAT. B-VMAT was on average superior for all other plan parameters. This is consistent with the findings by Fiandra et al. (19). **Figures 4E**, **F** show strong patient- and plan parameter dependences of differences between VMAT and B-VMAT.
- *VMAT vs. CP_x*: For small x, VMAT was clearly superior. For larger x, differences were dependent on plan parameter.
- *Breast*: Non-coplanar approaches scored best. B-VMAT was overall the clear winner, followed by NCP with 12 beams or more (NCP_x≥12). Superiority of B-VMAT could be related to geometrical constraints as defined by the butterfly geometry, limiting the dose delivered to the breasts.
- *Heart*: Non-coplanar approaches were best. NCP_x≥10 plans had on average a lower heart Dmean than B-VMAT. The superior heart sparing with NCP_15 and B-VMAT is illustrated for patient 3 in **Figure 3**.
- *Lung*: NCP_x≥13 was overall best for Dmean and V20Gy. B-VMAT was overall best for V5Gy but resulted in high V20Gy.
- *Low vs high dose in lungs and patient (V5Gy vs V20Gy)*: Compared to B-VMAT, NCP improved lung and patient V20Gy (mostly p < 0.001), at the cost of lungs and patient V5Gy (mostly p < 0.001) and breast V4Gy (only significant for right breast). This can also be observed in the dose distributions in **Figure 5**, where B-VMAT was less conformal around the tumor (red and yellow isodose lines), but showed less spread of low doses (light green and azure isodose lines in sagittal view), compared to CP_15 and NCP_15.
- *Dose conformality*: On average (**Figure 2**), conformality was best for VMAT (lowest CI), closely followed by NCP_15 and CP_15. B-VMAT was clearly the worst.
- *Overall observations:* In **Figure 4**, patients are sorted according to decreasing heart Dmean in NCP_15 plans. A clear reduction in differences among techniques is visible for patients with decreasing heart Dmean, showing a dependence on patient anatomy (**Figure B1 in Electronic Supplement B**) when selecting the optimal technique. E.g. patient 25 showed smaller differences between techniques, making the less complex CP or VMAT the favorable choice.

### Patient-Specific Beam Orientations

For NCP_15 and CP_15, patient group analyses were performed on selected beam directions. The population distributions of selected beam directions are shown in **Figure 5**. The rectangles in the left panel of **Figure 5** show the coplanar and non-coplanar beam directions used for B-VMAT. Non-coplanar beams resulting from a couch angle of 90° and gantry angles between 10° and 30°, entering the patient from anterior-inferior directions, were frequently present in NCP_15 plans. These entrance angles have a heart sparing/avoidance effect (see also sagittal views in **Figure 3**). The (couch, gantry) directions around (−70°, −30°) and around (−45°, −15°) were also often present in the NCP_15 plans. A clear prevalence of anterior beams was found in both NCP_15 and CP_15 with gantry angles between ±90°. For all patients, at least one anterior beam was present in the range −10° to 10° for CP_15 plans. Many beams in NCP_15 coincide with the anterior beam directions of B-VMAT. On the other hand, the posterior angles of B-VMAT were hardly selected in NCP_15. Apart from the clustered areas, **Figure 5** shows broad distributions of selected beam directions for NCP_15 and CP_15. This is in agreement with the large inter-patient variations in selected directions, shown in electronic appendix C.

## Discussion

To the best of our knowledge, in all published studies comparing beam configurations for treatment of mediastinal lymphoma patients, treatment plans were generated with manual trial-and-error planning, including selection of beam angles. It is well-known that manually generated plans may suffer from inter-and intra-planner quality variations, aggravated by the complex selection of optimal beam configurations. In this paper we present the first study using autoplanning with integrated beam angle optimization to systematically explore advantages and disadvantages of various coplanar and non-coplanar beam configuration approaches for young female mediastinal lymphoma patients. Due to this automation, plan generation became fully independent of planners, and the analyses could be based on a large number of high-quality plans.

From the 624 generated autoplans (26 patients with 24 autoplans), 617 (98.9%) satisfied the clinical PTV coverage requirement. The seven autoplans with insufficient PTV coverage were from the same patient (patient 0). Because of these plans with too low coverage, patient 0 was not included in patient population analyses comparing beam configuration approaches (see also *Results* section). Of the remaining 600 autoplans (25 patients with 24 autoplans), the dosimetric parameters compared favorably with those of corresponding clinically delivered plans, generated with manual plan generation. This observation was in agreement with the evaluations of 100 autoplans by the two physicians involved in this study who considered these plans of high quality (**Electronic Supplement A**).

There was not an overall superior beam configuration approach for the patient population, i.e. being on average best for all plan parameters. Performances of the various approaches were dependent on the considered OAR and the endpoint. There were also large inter-patient variations in the gain of one technique compared to another. However, overall there was a clear tendency towards improved plans with non-coplanar configurations (B-VMAT and NCP_x≥12). NCP_x≥12 was on average better in producing highly conformal plans with reduced high doses in the lungs and patient and also a reduced heart Dmean, while B-VMAT had reduced low-dose spread, related to the confinement of beam angles to the butterfly geometry. Levis et al. (36) have recently reported on a new-generation butterfly VMAT, where the coplanar part consists of a standard full-arc VMAT (FaB-VMAT). While this approach may solve some of the issues pointed out here for the B-VMAT approach (lack of conformity in the high doses), it might not be superior to NCP_15 for selected patients. In fact, the authors report a loss in breast dosimetry with FaB-VMAT for bulky tumors, compared to B-VMAT.

A distinct disadvantage of non-coplanar treatments can be an increase in delivery time. There is also enhanced risk of collisions due to human errors in delivery. Whether the dosimetrical benefit justifies increases in delivery time and complexity remains a clinical choice that may be highly dependent on the patient at hand with her specific plan quality improvements and required number of non-coplanar beams. In most radiotherapy departments, the number of ML patients is limited, which may render non-coplanar treatment (for a selected group) more feasible. Risks of collisions can be mitigated with adequate delivery protocols, and instruction and training of RTTs.

The observed large inter-patient variations in dosimetric differences between various beam set-ups are an incentive for prospective clinical use of automated planning to generate multiple plans for each new patient, and then select the best plan, considering quality and delivery time. This could further personalize radiotherapy for ML patients. We believe that for a clinical application, not all 24 autoplans discussed in this study need to be generated for each new patient. Coplanar plan generation could be limited to VMAT and for non-coplanar treatment, B-VMAT and, e.g., NCP_9 and NCP_15 could be generated. Based on a comparison of these plans, a final plan could be selected, or NCP_x plans with other beam numbers could be generated to refine the choice.

The seven autoplans of patient 0 with insufficient PTV coverage to avoid excessive OAR dose delivery were all coplanar with relatively low numbers of beams (5-11). For the remaining five coplanar plans with 12 to 15 beams and for all 12 non-coplanar plans, adequate coverage was obtained. Many of these plans also had superior OAR dose delivery compared to the clinical plan. The automated workflow presented above, based on automated generation of a small set of treatment plans for each patient, would naturally have avoided generation of the low beam number coplanar plans with unacceptably low PTV coverage.

This study and the proposed clinical workflow, based on generation of a small set of plans for each patient, are incentives for manufactures of treatment planning systems to extend their systems with advanced options for patient-specific beam angle optimization.

The automated planning applied in this study was developed to generate plans that balance all treatment aims in line with the clinical protocol. However, effectively, the various investigated beam angle approaches did in the end result in different overall balances between the objectives, resulting from the respective opportunities and limitations in beam angle choice (above). This could be extremely useful in case of co-morbidities or specific toxicity risks. E.g. for most patients with a heart comorbidity, NCP_x≥12 plans would be favorable, while B-VMAT would often be the modality of choice if low dose in the contralateral breast is of high relevance. Variations in plan quality could be further enhanced by also generating plans with wish-lists that focus on sparing of particular OARs. In a future work we will investigate pre-defined deviations from the clinical planning protocol, each focusing maximally on a specific endpoint/OAR.

In clinical planning, beam energies of 6, 10, and 18 MV were used, often also in combinations. For autoplanning in this study only 6 MV was used to avoid prolonged optimization times due to inclusion of beam energy optimization. Nevertheless, the obtained plan quality was high.

As mentioned in the M&M section, Erasmus-iCycle was used to optimize intensity profiles, i.e. time-consuming segmentation of the 600 plans was avoided. This does not impact the main conclusions of the paper; in many previous studies, we have demonstrated the ability to segment these plans for VMAT (35, 37, 38). Moreover, for the technique comparisons, only differences in plans were evaluated, and interesting differences were generally large.

We used heart Dmean for restricting the risk on radiation-induced cardiac toxicity. This is in line with the study by Darby et al. on radiation-induced cardiac toxicity in breast cancer patients (39). On the other hand, there are indications that selective sparing of heart substructures could be important (31, 40). To the best of our knowledge, there are no systematic studies for ML comparing planning with and without the use of heart substructures, including an evaluation of the impact on target dose and doses in the other OARs. Unfortunately, in our study these substructures were not delineated for clinical treatments, and were therefore not available for detailed analyses.

In research environments, different solutions for beam angle optimization have been proposed (33, 41–43). In our study, the solution developed by Breedveld et al. (33) has been used as shown to produce high quality results (37, 44). Comparisons of different algorithms are lacking.

In conclusion, using autoplanning including computerized coplanar and non-coplanar beam configuration optimization, 24 beam configuration approaches were compared for 25 young female mediastinal lymphoma patients. The quality of the applied autoplans was superior to that of manually generated, clinically delivered plans. Non-coplanar beam configurations were overall favorable, but significant patient-specific and plan-parameter-dependent dosimetric advantages and disadvantages of different beam configurations were observed, suggesting a need for prospective generation of multiple plans per patient to optimally personalize radiotherapy treatment. A workflow was proposed for automated generation of a small set of plans for each patient, followed by a selection.

## Data Availability Statement

All relevant data are within the paper and its supplementary files. Access to raw-data underlying the findings in this paper will be made possible on request to corresponding author.

## Ethics Statement

Ethical review and approval was not required for the study on human participants in accordance with the local legislation and institutional requirements. Written informed consent for participation was not required for this study in accordance with the national legislation and the institutional requirements.

## Author Contributions

LR and PC: conducting main research, brainstorming, and writing manuscript. JL: data, collection, brainstorming about research, and partial data analysis. JP and BH: supervision of research, brainstorming, and writing manuscript. SB: developing code and writing manuscript. MV and CJ: data collection, data revision, and writing manuscript. All authors contributed to the article and approved the submitted version.

## Funding

This work was in part funded by Elekta AB (Stockholm, Sweden). Erasmus MC Cancer Institute also has research collaborations with Accuray, Inc (Sunnyvale, USA) and Varian Medical Systems, Inc (Palo Alto, USA). The funder bodies were not involved in the study design, collection, analysis, interpretation of data, the writing of this article or the decision to submit it for publication.

## Conflict of Interest

The authors declare that the research was conducted in the absence of any commercial or financial relationships that could be construed as a potential conflict of interest.
