# Supplementary material for: On the Importance of Individualized, Non-Coplanar Beam Configurations in Mediastinal Lymphoma Radiotherapy, Optimized With Automated Planning
Source: Front Oncol. 2021 Apr 15;11:619929. doi: 10.3389/fonc.2021.619929 (PMC8082440; doi:10.3389/fonc.2021.619929)
Supplement: Supplementary file 1 [file Image_1.pdf]

Electronic supplement A

Quality of autoplans

Next to the analysis presented in the main body of the paper, two other types of analyses were performed to ensure that the autoplans used for beam angle comparisons were of high quality: detailed plan parameter comparisons between manually generated clinical plans (CLIN) and corresponding CLINauto plans and NCP_15 plans (section A1), and physicians' evaluations of autoplans considering full 3D dose distributions, DVHs, and DVH parameters (section A2).

Two-sided Wilcoxon signed-rank tests were used for statistical analyses with p-values lower than 0.05 indicating statistical significance in plan parameter differences.

A1. Comparison of CLIN plans with CLINauto and NCP_15 plans.

For the 20 patients that were clinically treated with IMRT (excluding patient 0, see paper body), the CLIN plan was compared to the CLINauto plan, i.e. an automatically generated IMRT plan with the same (manually selected) beam configuration as CLIN (Table A1). Significant improvements (statistically and clinically) with autoplanning were observed for PTV V95%, PTV V<90%, PTV V107%, lungs and patient V5Gy, and right breast V4Gy. On average, PTV V95% was 1.4% higher (maximum: 3.3%) and PTV underdose (V<90%) was 2 cc less (maximum: 10 cc). This came at the expense of slightly enhanced lung and patient V20Gy and CI, the latter clearly related with higher PTV coverage. Due to substantially improved PTV dose in the CLINauto plans, the latter plans were overall preferred over the CLIN plans.

The last columns in Table A1 show impressive plan quality improvements for NCP_15 compared to CLIN, related to the application of 15 non-coplanar IMRT beams with computer-optimized, individualized beam configurations, instead of 4-8 manually selected beams.

Figures A1 and A2 show for the individual patients, PTV and OAR plan parameters.

Table A1. Average and range (min, max) plan parameters for 20 clinical IMRT plans (CLIN), and differences with CLINauto (IMRT with same beam angles as CLIN) and with NCP_15 (IMRT with 15 beams with computer-optimized patient-specific beams). Colors indicate statistical significance (*p*<0.05), green: automation superior, red: CLIN superior, white: difference not significant.

Structure	Parameters	CLIN		CLINauto - CLIN		*p*	NCP_15 - CLIN		*p*
PTV	V95% (%)	98.2	(96.3, 99.7)	1.4	(0.1, 3.3)	<0.001	1.1	(-0.4, 3.1)	<0.001
	V<90% (cc)	2.2	(0, 10.6)	-2	(-10.4, 0.1)	<0.001	-1.5	(-9, 1.1)	0.040
	V107% (%)	1	(0, 2.6)	-0.8	(-2.3, 0.2)	<0.001	-0.8	(-2.3, 0.2)	<0.001
	CI	1.3	(1.1, 1.6)	0.2	(0, 0.3)	<0.001	-0.1	(-0.4, 0.1)	0.006
$Breast_R$	Dmean (Gy)	1.4	(0.1, 6.2)	-0.2	(-1, 0.6)	0.145	-0.4	(-1.6, 0.7)	0.033
	V4Gy (%)	8.1	(0, 35.4)	-2.4	(-10.2, 2.6)	0.011	-3.6	(-20.6, 4.6)	0.022
$Breast_L$	Dmean (Gy)	1.6	(0, 5.3)	-0.1	(-3, 0.5)	0.627	-0.4	(-3.4, 1)	0.145
	V4Gy (%)	8.9	(0, 37.6)	-1.7	(-27.5, 3.2)	0.379	-2	(-26.8, 7.8)	0.352
Heart	Dmean (Gy)	5.8	(0.2, 17.1)	0.1	(-1.8, 1.9)	0.391	-1.6	(-5.8, 1.7)	0.001
Lungs	Dmean (Gy)	8	(2.1, 14.3)	0	(-1.6, 1.2)	1.000	-1.6	(-3.3, 0)	<0.001
	V5Gy (%)	42.8	(10.6, 79.5)	-2.6	(-10.9, 2.8)	0.004	-9.6	(-23.9, 1.3)	<0.001
	V20Gy (%)	14.9	(3, 30)	2	(-1.3, 6.1)	<0.001	-3.4	(-8.5, 1.7)	<0.001
Patient	V5Gy (cc)	4665.7	(1192.9, 7917.9)	-187	(-950.8, 246.3)	0.033	-438.4	(-1777.8, 539.1)	0.007
	V20Gy (cc)	1665.5	(271.3, 4060.9)	323.3	(-3.7, 777.1)	<0.001	-221.6	(-900.5, 192.1)	0.009

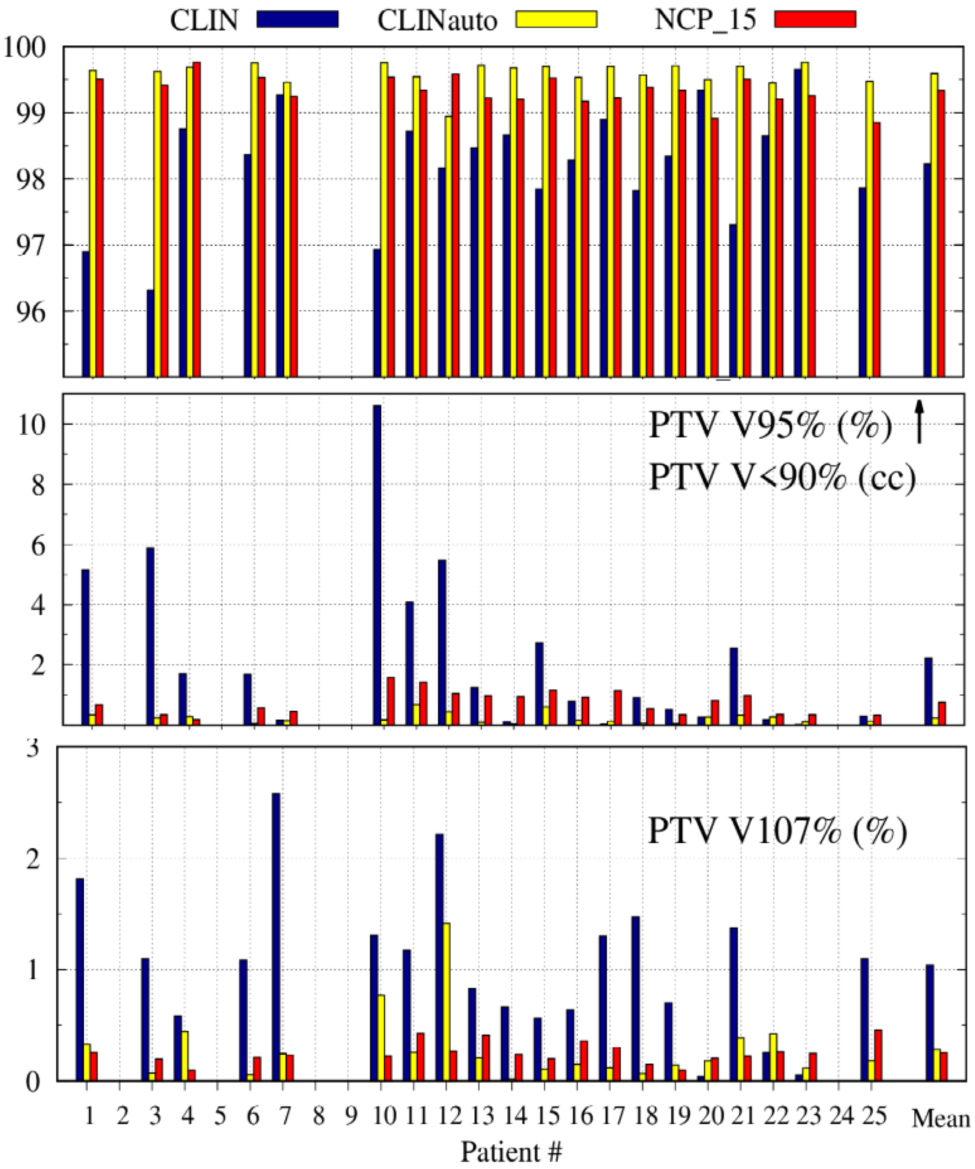

Figure A1. PTV plan parameters for 20 patients treated with IMRT: CLIN (blue), CLINauto (yellow) and NCP_15 (red).

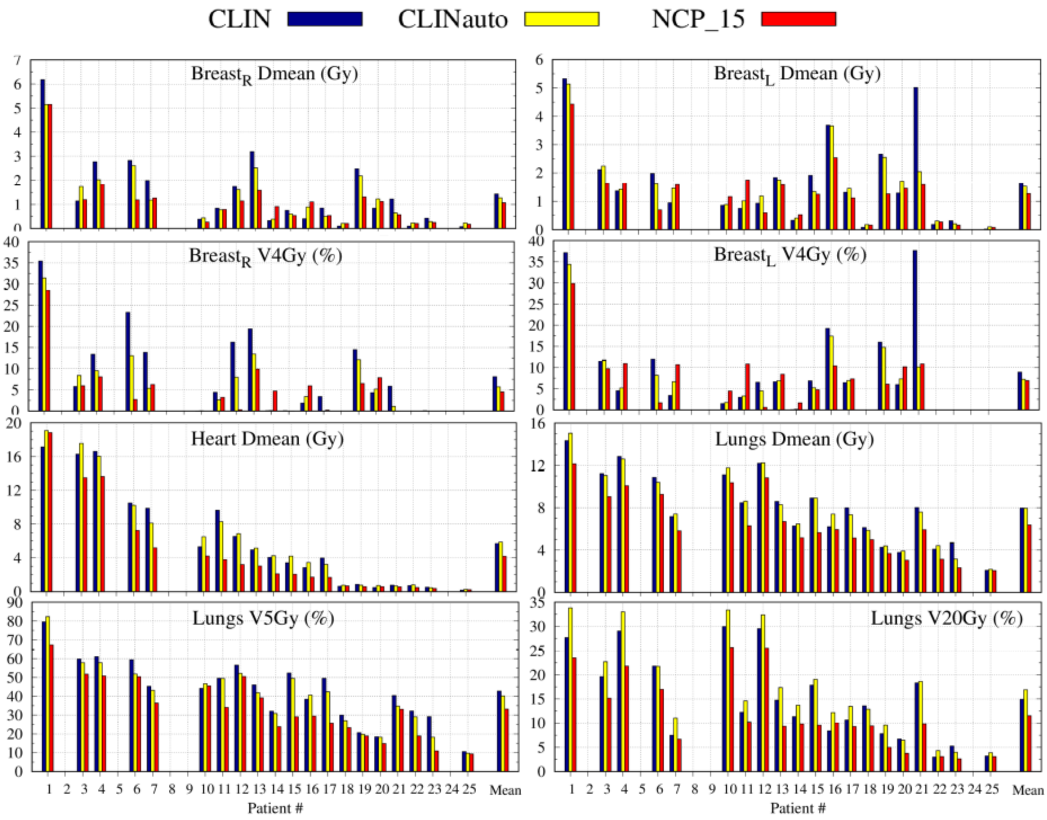

Figure A2. OAR plan parameters for 20 patients treated with IMRT: CLIN (blue), CLINauto (yellow) and NCP_15 (red).

A2. Physicians' evaluations of CP_9, NCP_15, VMAT, and B-VMAT autoplans

The in total 100 evaluated autoplans for patients 1-25 were considered of high quality by both physicians. For one physician, all evaluated autoplans were acceptable. In first instance, the other physician had doubts on 7 coplanar autoplans (3x CP_9, 4x VMAT) because of lungs V5Gy exceeding 55%. However, this parameter turned out to be in the range 61%-79.5% in the corresponding clinical plans and were accepted. The latter physician also had doubts on two non-coplanar plans with dose spread into the liver (resulting mean liver doses: 3.6 Gy and 2.5 Gy).
