# Supplementary material for: On the Importance of Individualized, Non-Coplanar Beam Configurations in Mediastinal Lymphoma Radiotherapy, Optimized With Automated Planning
Source: Front Oncol. 2021 Apr 15;11:619929. doi: 10.3389/fonc.2021.619929 (PMC8082440; doi:10.3389/fonc.2021.619929)
Supplement: Supplementary file 2 [file Image_2.pdf]

Electronic supplement B

B1 Patient cohort

Figure B1. Study patients ordered according to descending heart Dmean in the NCP_15 plans. Patient 0 was excluded from all analyses because of unacceptable autoplans (see Electronic appendix A). red: PTV, pink: patient.

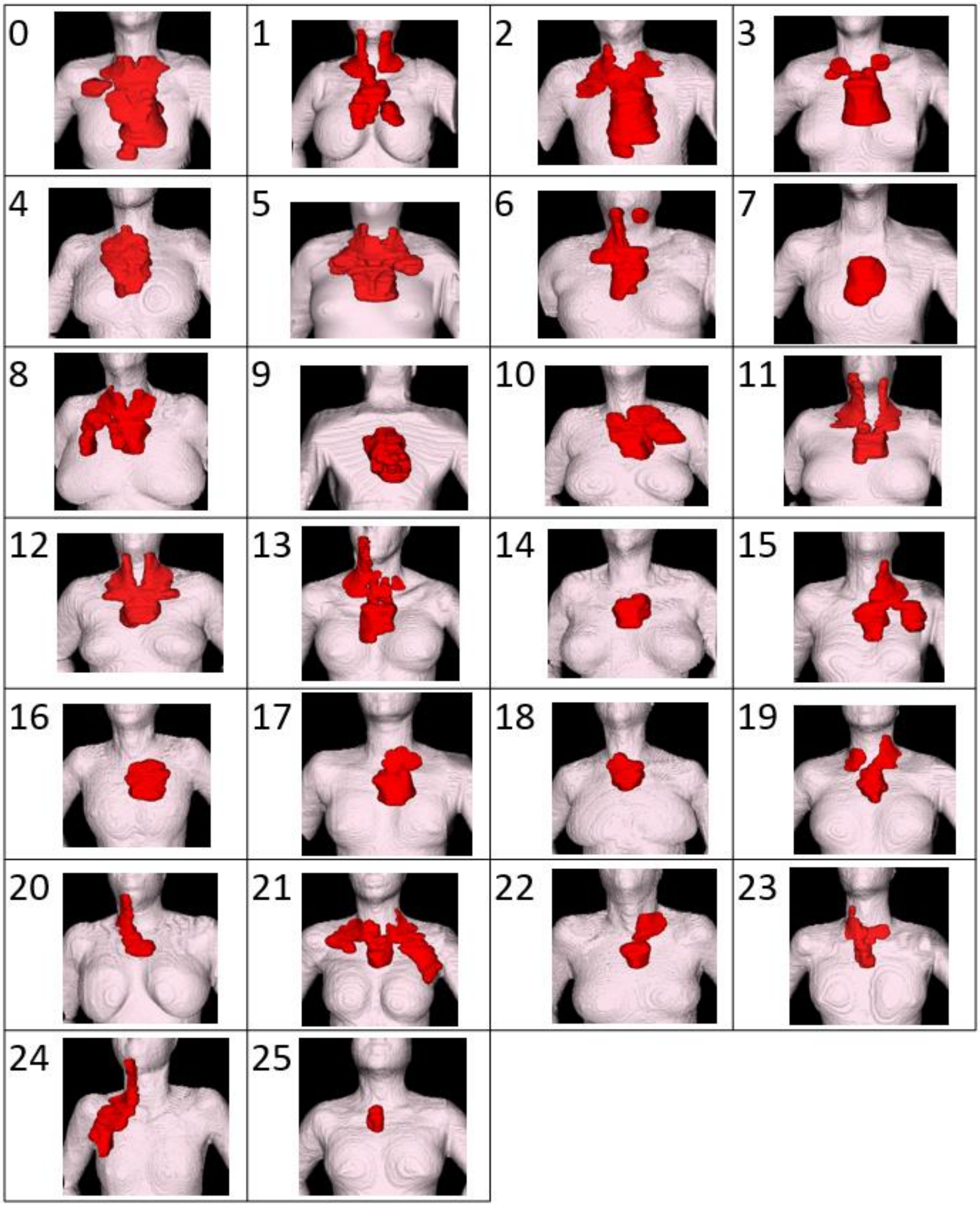

B2 Wish-list for mediastinal lymphoma autoplanning with Erasmus-iCycle

Autoplanning with Erasmus-iCycle is based on a patient-group-specific wish-list [1]. Hard constraints and prioritized objectives in the wish-list steer the optimizer in the multi-criterial plan generation. Constraints are always met, while objectives are goals that are optimized as much as possible, following the objective priorities and within the imposed hard constraints. Objectives are optimized sequentially, starting with the highest priority objective (priority 1). After each objective optimization, a constraint is added to the optimization problem, which is used for optimizing lower priority objectives without losing on obtained higher priority objective values [1-9].

The wish-list created in this study for young female mediastinal lymphoma patients, using the wish-list creation methodology as explained in the Electronic appendix of [8], is shown in Table B1. It contains constraints on (a) the PTV Dmean and Dmax to control tumor dose homogeneity, (b) shells at 0.3-5 cm distance from the PTV, to control dose fall off, (c) breast Dmean, and (d) entrance dose. With the LTCP cost function (logarithmic tumor control probability [9]) as first priority, plan generation always started with optimizing PTV coverage within the constraints, which was also clinically the most important objective. Next, the PTV minimum dose (priority 2) was optimized, again in line with clinical planning. Priority 3 tried to limit the maximum dose in a 2 cm ring around the PTV, while priority 4 aimed at reducing dose (to about 60% of the prescribed dose at 5 cm) in a larger volume, including the back muscles. With priorities 5-11, OAR doses were optimized, balancing dose delivery to breasts, heart and lungs. Mean doses and EUDs were used as cost functions. EUDs with a=0.5 were used to control the low-dose bath in lungs and breasts. As last objective, the dose at 1 cm from the PTV (shell) was reduced to further minimize dose outside the PTV where possible.

Table B1. Wish-list used in autoplanning for all patients and beam configurations. *: dose in first 2 cm inwards the patient contour, subtracting PTV expanded by 7 cm. **= PTV expanded with 2 cm – PTV, ***= patient – (PTV expanded by 5 cm). LTCP = Logarithmic tumor control probability, Dc = 95% * prescribed dose and α = cell sensitivity. EUD = Equivalent Uniform Dose, a = volume parameter. The use of goal and sufficient parameters is explained in [Breedveld 2012].

Constraints

Structure	Type	Limit
PTV	maximum	32.1 Gy
PTV	mean	30.6 Gy
Breast L	mean	5 Gy
Breast R	mean	5 Gy
Shell 3 mm from PTV	maximum	30 Gy
Shell 1 cm from PTV	maximum	28.5 Gy
Shell 3 cm from PTV	maximum	27 Gy
Shell 5 cm from PTV	maximum	22.5 Gy
Entrance dose*	maximum	18 Gy

Objectives

Priority	Structure	Type	Goal	Sufficient	Parameters
1	PTV	LTCP	0.2	0.2	D_c =28.5 Gy, α =0.8
2	PTV	minimum	28.5 Gy		
3	Ring 2 cm around PTV**	maximum	28.5 Gy		
4	Patient - PTV exp 5 cm***	maximum	21 Gy		
5	Lungs - PTV	EUD	6 Gy	6 Gy	a=0.5
6	Lungs - PTV	EUD	22 Gy	22 Gy	a=8
7	Breast L	EUD	0.9 Gy		a=0.5
7	Breast R	EUD	0.9 Gy		a=0.5
8	Heart - PTV	mean	0 Gy		
9	Lungs - PTV	mean	0 Gy		
10	Heart - PTV	EUD	0 Gy		a=8
11	Breast L	EUD	0 Gy		a=8
11	Breast R	EUD	0 Gy		a=8
12	Shell 1 cm from PTV	maximum	27 Gy		

B3 Mutual dosimetric comparisons of all 24 investigated beam configurations (related to Fig. 3 in the body of the manuscript)

Figure B2. Each table below presents mutual comparisons of all 24 beam configuration approaches for each of the evaluated dosimetric plan parameters of the autoplans. Above the diagonal, mean differences (Treatment A – Treatment B) for patients 1-25 are presented. Green colours point at statistically significant differences. On the diagonal (A=B), absolute population mean plan parameter values for all 24 beam configuration approaches are presented. Below the diagonal, P-values for the plan parameter differences, presented above the diagonal, are reported. * = p>0.05, green values = p<0.05

PTV V95% (%)

Treatment A \ Treatment B	CP_5	CP_6	CP_7	CP_8	CP_9	CP_{10}	CP_{11}	CP_{12}	CP_{13}	CP_{14}	CP_{15}	NCP_5	NCP_6	NCP_7	NCP_8	NCP_9	NCP_{10}	NCP_{11}	NCP_{12}	NCP_{13}	NCP_{14}	NCP_{15}	VMAT	B-VMAT
CP_5	99.6	-0.0	0.0	0.0	0.1	0.1	0.1	0.1	0.2	0.2	0.3	-0.1	-0.1	-0.0	0.0	0.1	0.1	0.1	0.1	0.2	0.2	0.2	0.2	0.2
CP_6	0.003	99.6	0.0	0.1	0.1	0.1	0.1	0.2	0.2	0.2	0.3	-0.1	-0.0	0.0	0.1	0.1	0.1	0.1	0.2	0.2	0.2	0.2	0.2	0.3
CP_7	0.001	0.001	99.6	0.0	0.1	0.1	0.1	0.1	0.1	0.2	0.2	-0.2	-0.1	-0.0	0.0	0.0	0.1	0.1	0.1	0.1	0.2	0.2	0.2	0.2
CP_8	<0.001	<0.001	0.002	99.6	0.0	0.0	0.1	0.1	0.1	0.1	0.2	-0.2	-0.1	-0.1	-0.0	0.0	0.0	0.1	0.1	0.1	0.1	0.2	0.2	0.2
CP_9	<0.001	<0.001	<0.001	<0.001	99.5	0.0	0.0	0.1	0.1	0.1	0.2	-0.2	-0.1	-0.1	-0.0	-0.0	0.0	0.0	0.1	0.1	0.1	0.1	0.1	0.2
CP_{10}	<0.001	<0.001	<0.001	<0.001	0.002	99.5	0.0	0.0	0.1	0.1	0.2	-0.2	-0.2	-0.1	-0.1	-0.0	-0.0	0.0	0.0	0.1	0.1	0.1	0.1	0.1
CP_{11}	<0.001	<0.001	<0.001	<0.001	<0.001	0.005	99.5	0.0	0.1	0.1	0.2	-0.2	-0.2	-0.1	-0.1	-0.0	-0.0	0.0	0.0	0.0	0.1	0.1	0.1	0.1
CP_{12}	<0.001	<0.001	<0.001	<0.001	<0.001	<0.001	<0.001	99.5	0.0	0.0	0.1	-0.3	-0.2	-0.1	-0.1	-0.1	-0.0	-0.0	0.0	0.0	0.1	0.1	0.1	0.1
CP_{13}	<0.001	<0.001	<0.001	<0.001	<0.001	<0.001	<0.001	<0.001	99.4	0.0	0.1	-0.3	-0.2	-0.2	-0.1	-0.1	-0.1	-0.1	-0.0	-0.0	0.0	0.1	0.1	0.1
CP_{14}	<0.001	<0.001	<0.001	<0.001	<0.001	<0.001	<0.001	<0.001	0.028	99.4	0.1	-0.3	-0.2	-0.2	-0.1	-0.1	-0.1	-0.1	-0.0	-0.0	0.0	0.0	0.0	0.1
CP_{15}	<0.001	<0.001	<0.001	<0.001	<0.001	<0.001	<0.001	<0.001	<0.001	<0.001	99.3	-0.4	-0.3	-0.3	-0.2	-0.2	-0.2	-0.2	-0.1	-0.1	-0.1	-0.0	-0.0	-0.0
NCP_5	*	<0.001	<0.001	<0.001	<0.001	<0.001	<0.001	<0.001	<0.001	<0.001	<0.001	99.7	0.1	0.1	0.2	0.2	0.2	0.2	0.3	0.3	0.3	0.3	0.3	0.4
NCP_6	*	*	<0.001	<0.001	<0.001	<0.001	<0.001	<0.001	<0.001	<0.001	<0.001	<0.001	99.7	0.1	0.1	0.1	0.2	0.2	0.2	0.2	0.3	0.3	0.3	0.3
NCP_7	0.008	*	*	0.012	0.005	<0.001	<0.001	<0.001	<0.001	<0.001	<0.001	<0.001	<0.001	99.6	0.0	0.1	0.1	0.1	0.1	0.2	0.2	0.2	0.2	0.2
NCP_8	0.001	0.015	*	*	*	0.009	0.005	0.001	<0.001	<0.001	<0.001	<0.001	<0.001	<0.001	99.6	0.0	0.0	0.1	0.1	0.1	0.1	0.2	0.2	0.2
NCP_9	<0.001	0.005	0.020	*	*	*	0.045	0.006	<0.001	<0.001	<0.001	<0.001	<0.001	<0.001	<0.001	99.5	0.0	0.0	0.1	0.1	0.1	0.1	0.1	0.2
NCP_{10}	<0.001	0.002	0.004	*	*	*	*	0.019	0.001	<0.001	<0.001	<0.001	<0.001	<0.001	<0.001	0.002	99.5	0.0	0.0	0.1	0.1	0.1	0.1	0.1
NCP_{11}	<0.001	0.002	<0.001	0.009	*	*	*	*	0.024	0.009	0.002	<0.001	<0.001	<0.001	<0.001	<0.001	<0.001	99.5	0.0	0.0	0.1	0.1	0.1	0.1
NCP_{12}	<0.001	<0.001	<0.001	0.001	0.009	*	*	*	*	*	0.011	<0.001	<0.001	<0.001	<0.001	<0.001	<0.001	<0.001	99.5	0.0	0.1	0.1	0.1	0.1
NCP_{13}	<0.001	<0.001	<0.001	<0.001	<0.001	0.008	0.025	*	*	*	0.037	<0.001	<0.001	<0.001	<0.001	<0.001	<0.001	<0.001	<0.001	99.4	0.0	0.1	0.1	0.1
NCP_{14}	<0.001	<0.001	<0.001	<0.001	<0.001	<0.001	0.002	0.015	*	*	*	<0.001	<0.001	<0.001	<0.001	<0.001	<0.001	<0.001	<0.001	<0.001	99.4	0.0	0.0	0.0
NCP_{15}	<0.001	<0.001	<0.001	<0.001	<0.001	<0.001	<0.001	<0.001	0.022	*	*	<0.001	<0.001	<0.001	<0.001	<0.001	<0.001	<0.001	<0.001	<0.001	<0.001	99.4	-0.0	0.0
VMAT	<0.001	<0.001	<0.001	<0.001	<0.001	<0.001	<0.001	<0.001	0.003	0.007	*	<0.001	<0.001	<0.001	<0.001	<0.001	<0.001	0.001	0.006	0.014	*	*	99.4	0.0
B-VMAT	<0.001	<0.001	<0.001	<0.001	<0.001	<0.001	<0.001	0.005	0.028	*	*	<0.001	<0.001	<0.001	<0.001	<0.001	<0.001	<0.001	<0.001	0.006	*	*	*	99.4

PTV V90% (cc)

Treatment A \ Treatment B	CP_5	CP_6	CP_7	CP_8	CP_9	CP_{10}	CP_{11}	CP_{12}	CP_{13}	CP_{14}	CP_{15}	NCP_5	NCP_6	NCP_7	NCP_8	NCP_9	NCP_{10}	NCP_{11}	NCP_{12}	NCP_{13}	NCP_{14}	NCP_{15}	VMAT	B-VMAT
CP_5	689.3	-0.1	-0.1	-0.0	0.0	0.1	0.1	0.1	0.2	0.2	0.2	-0.2	-0.1	-0.1	0.0	0.1	0.1	0.2	0.2	0.2	0.3	0.3	0.2	0.4
CP_6	0.001	689.4	0.1	0.1	0.2	0.2	0.2	0.3	0.3	0.3	0.4	-0.1	0.0	0.1	0.1	0.2	0.3	0.3	0.3	0.4	0.4	0.4	0.3	0.5
CP_7	<0.001	0.001	689.4	0.1	0.1	0.2	0.2	0.2	0.2	0.3	0.3	-0.1	-0.1	0.0	0.1	0.2	0.2	0.2	0.3	0.3	0.4	0.4	0.3	0.5
CP_8	<0.001	<0.001	<0.001	689.3	0.0	0.1	0.1	0.1	0.2	0.2	0.2	-0.2	-0.1	-0.0	0.0	0.1	0.1	0.2	0.2	0.3	0.3	0.3	0.2	0.4
CP_9	<0.001	<0.001	<0.001	0.003	689.3	0.1	0.1	0.1	0.1	0.2	0.2	-0.3	-0.2	-0.1	-0.0	0.1	0.1	0.1	0.2	0.2	0.2	0.3	0.2	0.4
CP_{10}	<0.001	<0.001	<0.001	<0.001	<0.001	689.2	0.0	0.1	0.1	0.1	0.2	-0.3	-0.2	-0.1	-0.1	0.0	0.0	0.1	0.1	0.2	0.2	0.2	0.1	0.3
CP_{11}	<0.001	<0.001	<0.001	<0.001	<0.001	0.028	689.2	0.0	0.1	0.1	0.1	-0.3	-0.2	-0.2	-0.1	-0.0	0.0	0.1	0.1	0.1	0.2	0.2	0.1	0.3
CP_{12}	<0.001	<0.001	<0.001	<0.001	<0.001	0.002	0.004	689.2	0.0	0.1	0.1	-0.4	-0.3	-0.2	-0.1	-0.1	-0.0	0.0	0.1	0.1	0.1	0.2	0.0	0.3
CP_{13}	<0.001	<0.001	<0.001	<0.001	<0.001	<0.001	<0.001	<0.001	689.1	0.0	0.1	-0.4	-0.3	-0.2	-0.2	-0.1	-0.1	-0.0	0.0	0.1	0.1	0.1	0.0	0.2
CP_{14}	<0.001	<0.001	<0.001	<0.001	<0.001	<0.001	<0.001	<0.001	0.022	689.1	0.0	-0.4	-0.3	-0.3	-0.2	-0.1	-0.1	-0.0	-0.0	0.1	0.1	0.1	-0.0	0.2
CP_{15}	<0.001	<0.001	<0.001	<0.001	<0.001	<0.001	<0.001	<0.001	<0.001	0.002	689.1	-0.5	-0.4	-0.3	-0.2	-0.1	-0.1	-0.1	-0.0	0.0	0.0	0.1	-0.1	0.2
NCP_5	*	0.048	<0.001	<0.001	<0.001	<0.001	<0.001	<0.001	<0.001	<0.001	<0.001	689.5	0.1	0.2	0.2	0.3	0.3	0.4	0.4	0.5	0.5	0.5	0.4	0.6
NCP_6	0.024	*	*	0.049	0.007	<0.001	<0.001	<0.001	<0.001	<0.001	<0.001	<0.001	689.4	0.1	0.1	0.2	0.3	0.3	0.3	0.4	0.4	0.4	0.3	0.5
NCP_7	<0.001	0.037	*	*	*	0.015	0.004	<0.001	<0.001	<0.001	<0.001	<0.001	<0.001	689.4	0.1	0.1	0.2	0.2	0.2	0.3	0.3	0.4	0.2	0.5
NCP_8	<0.001	0.012	*	*	*	*	*	0.044	0.014	0.008	<0.001	<0.001	<0.001	0.002	689.3	0.1	0.1	0.2	0.2	0.2	0.3	0.3	0.2	0.4
NCP_9	<0.001	0.001	0.003	*	*	*	*	*	*	*	0.015	<0.001	<0.001	<0.001	<0.001	689.2	0.0	0.1	0.1	0.2	0.2	0.2	0.1	0.3
NCP_{10}	<0.001	<0.001	0.002	0.021	*	*	*	*	*	*	0.044	<0.001	<0.001	<0.001	<0.001	<0.001	689.2	0.0	0.1	0.1	0.2	0.2	0.1	0.3
NCP_{11}	<0.001	<0.001	<0.001	0.006	0.022	*	*	*	*	*	*	<0.001	<0.001	<0.001	<0.001	<0.001	<0.001	689.1	0.0	0.1	0.1	0.1	0.0	0.2
NCP_{12}	<0.001	<0.001	<0.001	0.002	0.009	*	*	*	*	*	*	<0.001	<0.001	<0.001	<0.001	<0.001	<0.001	0.025	689.1	0.1	0.1	0.1	-0.0	0.2
NCP_{13}	<0.001	<0.001	<0.001	<0.001	0.001	0.011	0.014	*	*	*	*	<0.001	<0.001	<0.001	<0.001	<0.001	<0.001	<0.001	<0.001	689.1	0.0	0.1	-0.1	0.2
NCP_{14}	<0.001	<0.001	<0.001	<0.001	0.001	0.006	0.011	0.032	*	*	*	<0.001	<0.001	<0.001	<0.001	<0.001	<0.001	<0.001	<0.001	0.006	689.0	0.0	-0.1	0.1
NCP_{15}	<0.001	<0.001	<0.001	<0.001	<0.001	0.002	0.003	0.010	0.028	*	*	<0.001	<0.001	<0.001	<0.001	<0.001	<0.001	<0.001	<0.001	<0.001	0.010	689.0	-0.1	0.1
VMAT	<0.001	<0.001	<0.001	<0.001	<0.001	0.022	0.018	*	*	*	*	<0.001	<0.001	<0.001	0.001	0.039	*	*	*	*	0.037	0.007	689.1	0.2
B-VMAT	<0.001	<0.001	<0.001	<0.001	<0.001	<0.001	0.001	0.003	0.007	0.013	0.026	<0.001	<0.001	<0.001	<0.001	<0.001	<0.001	0.007	0.014	*	*	*	0.005	688.9

PTV V107% (%)

Treatment B (columns) / **Treatment A** (rows)

	CP_{5}	CP_{6}	CP_{7}	CP_{8}	CP_{9}	CP_{10}	CP_{11}	CP_{12}	CP_{13}	CP_{14}	CP_{15}	NCP_{5}	NCP_{6}	NCP_{7}	NCP_{8}	NCP_{9}	NCP_{10}	NCP_{11}	NCP_{12}	NCP_{13}	NCP_{14}	NCP_{15}	VMAT	B-VMAT
CP_{5}	0.4	0.1	0.1	0.1	0.1	0.1	0.1	0.1	0.1	0.1	0.0	0.2	0.2	0.2	0.2	0.2	0.2	0.2	0.2	0.1	0.1	0.1	0.1	0.1
CP_{6}	*	0.2	-0.0	-0.0	-0.0	-0.0	-0.0	-0.0	-0.1	-0.1	-0.1	0.0	0.1	0.1	0.1	0.0	0.0	0.0	0.0	0.0	-0.0	-0.0	-0.0	-0.1
CP_{7}	*	0.017	0.2	-0.0	0.0	0.0	-0.0	-0.0	-0.0	-0.1	-0.1	0.1	0.1	0.1	0.1	0.0	0.0	0.0	0.0	0.0	0.0	-0.0	-0.0	-0.1
CP_{8}	0.038	0.006	*	0.3	0.0	0.0	-0.0	-0.0	-0.0	-0.1	-0.1	0.1	0.1	0.1	0.1	0.1	0.1	0.0	0.0	0.0	0.0	0.0	-0.0	-0.0
CP_{9}	*	0.016	*	*	0.2	-0.0	-0.0	-0.0	-0.0	-0.1	-0.1	0.0	0.1	0.1	0.1	0.0	0.0	0.0	0.0	0.0	0.0	-0.0	-0.0	-0.1
CP_{10}	0.021	0.004	*	*	*	0.2	-0.0	-0.0	-0.0	-0.1	-0.1	0.1	0.1	0.1	0.1	0.0	0.0	0.0	0.0	0.0	0.0	-0.0	-0.0	-0.1
CP_{11}	0.013	0.005	*	*	*	*	0.3	-0.0	-0.0	-0.0	-0.1	0.1	0.1	0.1	0.1	0.1	0.1	0.0	0.1	0.0	0.0	0.0	-0.0	-0.0
CP_{12}	0.006	<0.001	0.013	*	0.016	0.006	*	0.3	-0.0	-0.0	-0.1	0.1	0.1	0.1	0.1	0.1	0.1	0.1	0.1	0.0	0.0	0.0	0.0	-0.0
CP_{13}	0.008	<0.001	0.001	0.013	0.004	0.002	0.031	*	0.3	-0.0	-0.1	0.1	0.1	0.1	0.1	0.1	0.1	0.1	0.1	0.1	0.1	0.0	0.0	-0.0
CP_{14}	0.004	<0.001	<0.001	0.009	0.002	<0.001	0.003	0.001	0.019	0.3	-0.1	0.1	0.1	0.1	0.1	0.1	0.1	0.1	0.1	0.1	0.1	0.1	0.0	0.0
CP_{15}	0.003	<0.001	<0.001	0.002	<0.001	<0.001	<0.001	0.002	0.002	*	0.4	0.2	0.2	0.2	0.2	0.2	0.2	0.1	0.2	0.1	0.1	0.1	0.1	0.1
NCP_{5}	*	*	0.044	0.011	*	0.042	0.023	0.012	0.007	0.005	0.003	0.2	0.0	0.0	0.0	-0.0	-0.0	-0.0	-0.0	-0.0	-0.0	-0.1	-0.1	-0.1
NCP_{6}	*	*	0.004	0.003	0.006	<0.001	<0.001	<0.001	<0.001	<0.001	<0.001	*	0.2	-0.0	-0.0	-0.0	-0.0	-0.0	-0.0	-0.1	-0.1	-0.1	-0.1	-0.1
NCP_{7}	*	*	0.048	0.010	0.013	<0.001	<0.001	<0.001	<0.001	<0.001	<0.001	*	*	0.2	0.0	-0.0	-0.0	-0.0	-0.0	-0.0	-0.1	-0.1	-0.1	-0.1
NCP_{8}	*	*	0.033	0.010	0.024	0.002	0.001	<0.001	<0.001	<0.001	<0.001	*	*	*	0.2	-0.0	-0.0	-0.0	-0.0	-0.1	-0.1	-0.1	-0.1	-0.1
NCP_{9}	*	*	*	*	*	*	0.027	0.005	<0.001	<0.001	<0.001	*	*	*	0.016	0.2	-0.0	-0.0	-0.0	-0.0	-0.0	-0.0	-0.1	-0.1
NCP_{10}	*	*	*	*	*	*	0.015	<0.001	<0.001	<0.001	<0.001	*	*	*	*	*	0.2	-0.0	-0.0	-0.0	-0.0	-0.0	-0.1	-0.1
NCP_{11}	*	*	*	*	*	*	*	0.014	<0.001	<0.001	<0.001	*	0.003	0.008	<0.001	*	0.022	0.2	0.0	-0.0	-0.0	-0.0	-0.1	-0.1
NCP_{12}	*	*	*	*	*	*	*	0.016	0.001	<0.001	<0.001	*	0.014	0.006	0.007	*	*	*	0.2	-0.0	-0.0	-0.0	-0.1	-0.1
NCP_{13}	*	*	*	*	*	*	*	*	0.008	0.001	<0.001	*	0.004	0.003	<0.001	0.014	0.002	*	*	0.2	-0.0	-0.0	-0.0	-0.1
NCP_{14}	*	*	*	*	*	*	*	*	0.026	0.006	0.003	*	<0.001	0.001	<0.001	0.004	<0.001	0.016	0.009	*	0.2	-0.0	-0.0	-0.1
NCP_{15}	*	0.038	*	*	*	*	*	*	*	0.034	0.018	*	<0.001	0.002	<0.001	0.002	<0.001	0.020	0.009	0.007	*	0.2	-0.0	-0.1
VMAT	0.012	0.003	0.009	*	*	*	*	*	*	0.011	0.004	0.030	<0.001	<0.001	<0.001	0.002	<0.001	0.005	0.018	0.043	*	*	0.3	-0.0
B-VMAT	0.001	<0.001	0.004	0.036	0.013	0.012	*	*	*	*	*	0.001	<0.001	<0.001	<0.001	<0.001	<0.001	0.002	0.002	0.002	0.012	0.021	*	0.3

CI

Treatment B (columns) / **Treatment A** (rows)

	CP_{5}	CP_{6}	CP_{7}	CP_{8}	CP_{9}	CP_{10}	CP_{11}	CP_{12}	CP_{13}	CP_{14}	CP_{15}	NCP_{5}	NCP_{6}	NCP_{7}	NCP_{8}	NCP_{9}	NCP_{10}	NCP_{11}	NCP_{12}	NCP_{13}	NCP_{14}	NCP_{15}	VMAT	B-VMAT
CP_{5}	1.4	0.0	0.1	0.1	0.1	0.1	0.1	0.2	0.2	0.2	0.2	-0.0	0.0	0.1	0.1	0.1	0.1	0.2	0.2	0.2	0.2	0.2	0.2	0.0
CP_{6}	<0.001	1.4	0.0	0.0	0.1	0.1	0.1	0.1	0.1	0.1	0.2	-0.1	-0.0	0.0	0.0	0.1	0.1	0.1	0.1	0.2	0.2	0.2	0.2	-0.0
CP_{7}	<0.001	<0.001	1.4	0.0	0.0	0.1	0.1	0.1	0.1	0.1	0.1	-0.1	-0.0	-0.0	0.0	0.0	0.1	0.1	0.1	0.1	0.1	0.2	0.2	-0.0
CP_{8}	<0.001	<0.001	<0.001	1.3	0.0	0.0	0.1	0.1	0.1	0.1	0.1	-0.1	-0.1	-0.0	0.0	0.0	0.1	0.1	0.1	0.1	0.1	0.1	0.1	-0.1
CP_{9}	<0.001	<0.001	<0.001	<0.001	1.3	0.0	0.0	0.0	0.1	0.1	0.1	-0.1	-0.1	-0.1	-0.0	0.0	0.0	0.1	0.1	0.1	0.1	0.1	0.1	-0.1
CP_{10}	<0.001	<0.001	<0.001	<0.001	<0.001	1.3	0.0	0.0	0.0	0.1	0.1	-0.1	-0.1	-0.1	-0.0	-0.0	0.0	0.0	0.0	0.1	0.1	0.1	0.1	-0.1
CP_{11}	<0.001	<0.001	<0.001	<0.001	<0.001	<0.001	1.3	0.0	0.0	0.0	0.1	-0.2	-0.1	-0.1	-0.1	-0.0	-0.0	0.0	0.0	0.1	0.1	0.1	0.1	-0.1
CP_{12}	<0.001	<0.001	<0.001	<0.001	<0.001	<0.001	<0.001	1.3	0.0	0.0	0.0	-0.2	-0.1	-0.1	-0.1	-0.0	-0.0	0.0	0.0	0.0	0.1	0.1	0.1	-0.1
CP_{13}	<0.001	<0.001	<0.001	<0.001	<0.001	<0.001	<0.001	<0.001	1.2	0.0	0.0	-0.2	-0.2	-0.1	-0.1	-0.1	-0.0	-0.0	0.0	0.0	0.0	0.1	0.1	-0.1
CP_{14}	<0.001	<0.001	<0.001	<0.001	<0.001	<0.001	<0.001	<0.001	<0.001	1.2	0.0	-0.2	-0.2	-0.1	-0.1	-0.1	-0.0	-0.0	-0.0	0.0	0.0	0.0	0.0	-0.2
CP_{15}	<0.001	<0.001	<0.001	<0.001	<0.001	<0.001	<0.001	<0.001	<0.001	<0.001	1.2	-0.2	-0.2	-0.1	-0.1	-0.1	-0.1	-0.0	-0.0	-0.0	0.0	0.0	0.0	-0.2
NCP_{5}	0.006	<0.001	<0.001	<0.001	<0.001	<0.001	<0.001	<0.001	<0.001	<0.001	<0.001	1.4	0.0	0.1	0.1	0.1	0.2	0.2	0.2	0.2	0.2	0.2	0.3	0.1
NCP_{6}	0.006	0.009	<0.001	<0.001	<0.001	<0.001	<0.001	<0.001	<0.001	<0.001	<0.001	<0.001	1.4	0.0	0.1	0.1	0.1	0.1	0.2	0.2	0.2	0.2	0.2	0.0
NCP_{7}	<0.001	*	*	<0.001	<0.001	<0.001	<0.001	<0.001	<0.001	<0.001	<0.001	<0.001	<0.001	1.4	0.0	0.1	0.1	0.1	0.1	0.1	0.2	0.2	0.2	-0.0
NCP_{8}	<0.001	<0.001	0.002	*	0.005	<0.001	<0.001	<0.001	<0.001	<0.001	<0.001	<0.001	<0.001	<0.001	1.3	0.0	0.1	0.1	0.1	0.1	0.1	0.1	0.1	-0.1
NCP_{9}	<0.001	<0.001	<0.001	<0.001	*	0.020	0.001	<0.001	<0.001	<0.001	<0.001	<0.001	<0.001	<0.001	<0.001	1.3	0.0	0.0	0.1	0.1	0.1	0.1	0.1	-0.1
NCP_{10}	<0.001	<0.001	<0.001	<0.001	<0.001	*	*	0.010	<0.001	<0.001	<0.001	<0.001	<0.001	<0.001	<0.001	<0.001	1.3	0.0	0.0	0.1	0.1	0.1	0.1	-0.1
NCP_{11}	<0.001	<0.001	<0.001	<0.001	<0.001	<0.001	*	*	*	<0.001	<0.001	<0.001	<0.001	<0.001	<0.001	<0.001	<0.001	1.3	0.0	0.0	0.0	0.1	0.1	-0.1
NCP_{12}	<0.001	<0.001	<0.001	<0.001	<0.001	<0.001	<0.001	0.011	*	*	<0.001	<0.001	<0.001	<0.001	<0.001	<0.001	<0.001	<0.001	1.2	0.0	0.0	0.0	0.1	-0.1
NCP_{13}	<0.001	<0.001	<0.001	<0.001	<0.001	<0.001	<0.001	<0.001	0.001	*	*	<0.001	<0.001	<0.001	<0.001	<0.001	<0.001	<0.001	<0.001	1.2	0.0	0.0	0.0	-0.2
NCP_{14}	<0.001	<0.001	<0.001	<0.001	<0.001	<0.001	<0.001	<0.001	<0.001	<0.001	0.016	<0.001	<0.001	<0.001	<0.001	<0.001	<0.001	<0.001	<0.001	<0.001	1.2	0.0	0.0	-0.2
NCP_{15}	<0.001	<0.001	<0.001	<0.001	<0.001	<0.001	<0.001	<0.001	<0.001	<0.001	<0.001	<0.001	<0.001	<0.001	<0.001	<0.001	<0.001	<0.001	<0.001	<0.001	<0.001	1.2	0.0	-0.2
VMAT	<0.001	<0.001	<0.001	<0.001	<0.001	<0.001	<0.001	<0.001	<0.001	<0.001	<0.001	<0.001	<0.001	<0.001	<0.001	<0.001	<0.001	<0.001	<0.001	<0.001	<0.001	0.017	1.2	-0.2
B-VMAT	0.023	*	0.006	<0.001	<0.001	<0.001	<0.001	<0.001	<0.001	<0.001	<0.001	<0.001	*	*	<0.001	<0.001	<0.001	<0.001	<0.001	<0.001	<0.001	<0.001	<0.001	1.4

Right Breast Dmean (Gy)

Treatment B (columns); Treatment A (rows)

	CP_5	CP_6	CP_7	CP_8	CP_9	CP_{10}	CP_{11}	CP_{12}	CP_{13}	CP_{14}	CP_{15}	NCP_5	NCP_6	NCP_7	NCP_8	NCP_9	NCP_{10}	NCP_{11}	NCP_{12}	NCP_{13}	NCP_{14}	NCP_{15}	VMAT	B-VMAT
CP_5	1.8	-0.0	0.0	0.0	0.0	0.0	0.1	0.1	0.1	0.1	0.1	-0.0	0.0	0.1	0.1	0.2	0.2	0.2	0.2	0.2	0.2	0.2	0.1	0.4
CP_6	*	1.8	0.0	0.0	0.0	0.0	0.1	0.1	0.1	0.1	0.1	-0.0	0.0	0.1	0.2	0.2	0.2	0.2	0.3	0.3	0.3	0.2	0.1	0.4
CP_7	*	*	1.8	0.0	0.0	0.0	0.0	0.0	0.1	0.1	0.1	-0.0	-0.0	0.1	0.1	0.1	0.2	0.2	0.2	0.2	0.2	0.2	0.1	0.3
CP_8	*	*	*	1.8	0.0	0.0	0.0	0.0	0.0	0.1	0.1	-0.0	-0.0	0.1	0.1	0.1	0.2	0.2	0.2	0.2	0.2	0.2	0.1	0.3
CP_9	*	0.033	0.030	*	1.8	0.0	0.0	0.0	0.0	0.0	0.0	-0.1	-0.0	0.1	0.1	0.1	0.1	0.2	0.2	0.2	0.2	0.2	0.1	0.3
CP_{10}	*	*	*	*	*	1.8	0.0	0.0	0.0	0.0	0.0	-0.1	-0.0	0.1	0.1	0.1	0.1	0.2	0.2	0.2	0.2	0.2	0.1	0.3
CP_{11}	*	0.036	*	0.040	*	*	1.8	0.0	0.0	0.0	0.0	-0.1	-0.0	0.0	0.1	0.1	0.1	0.2	0.2	0.2	0.2	0.2	0.0	0.3
CP_{12}	*	0.041	*	*	*	*	*	1.8	0.0	0.0	0.0	-0.1	-0.0	0.0	0.1	0.1	0.1	0.2	0.2	0.2	0.2	0.2	0.0	0.3
CP_{13}	0.048	0.034	0.020	0.029	0.046	0.015	*	*	1.8	0.0	0.0	-0.1	-0.1	0.0	0.1	0.1	0.1	0.2	0.2	0.2	0.2	0.2	0.0	0.3
CP_{14}	0.025	0.020	0.037	*	*	*	*	*	*	1.8	0.0	-0.1	-0.1	0.0	0.1	0.1	0.1	0.1	0.2	0.2	0.2	0.2	0.0	0.3
CP_{15}	0.021	0.006	0.027	*	*	*	*	*	*	*	1.8	-0.1	-0.1	0.0	0.1	0.1	0.1	0.1	0.2	0.2	0.2	0.2	0.0	0.3
NCP_5	*	*	*	*	*	*	*	*	*	*	*	1.9	0.0	0.1	0.2	0.2	0.2	0.3	0.3	0.3	0.3	0.3	0.1	0.4
NCP_6	*	*	*	*	*	*	*	*	*	*	*	*	1.8	0.1	0.1	0.1	0.2	0.2	0.2	0.2	0.2	0.2	0.1	0.3
NCP_7	*	*	*	*	*	*	*	*	*	*	*	0.018	0.003	1.7	0.1	0.1	0.1	0.1	0.2	0.2	0.2	0.1	0.0	0.3
NCP_8	*	0.045	0.033	0.032	*	*	*	*	*	*	*	0.019	<0.001	0.039	1.7	0.0	0.0	0.1	0.1	0.1	0.1	0.1	-0.0	0.2
NCP_9	0.030	0.011	0.008	0.011	0.016	0.037	*	*	*	*	*	0.021	0.006	*	*	1.7	0.0	0.1	0.1	0.1	0.1	0.1	-0.1	0.2
NCP_{10}	0.019	0.012	0.006	0.012	0.012	0.028	*	*	*	*	*	0.019	0.005	*	*	*	1.6	0.0	0.1	0.1	0.1	0.0	-0.1	0.2
NCP_{11}	0.005	0.002	0.002	0.003	0.005	0.007	0.010	0.021	0.032	*	*	0.005	0.001	0.004	0.022	0.002	<0.001	1.6	0.0	0.0	0.0	0.0	-0.1	0.1
NCP_{12}	0.006	0.001	0.002	0.003	0.003	0.004	0.007	0.011	0.016	0.025	0.018	0.005	<0.001	0.012	0.036	0.007	0.002	*	1.6	-0.0	-0.0	-0.0	-0.1	0.1
NCP_{13}	0.006	0.002	0.003	0.004	0.006	0.007	0.012	0.022	0.031	0.046	0.025	0.003	<0.001	0.008	0.030	0.023	0.024	*	*	1.6	0.0	-0.0	-0.1	0.1
NCP_{14}	0.006	0.001	0.002	0.003	0.004	0.003	0.006	0.010	0.013	0.015	0.010	0.004	<0.001	0.016	*	0.008	0.026	*	*	*	1.6	-0.0	-0.1	0.1
NCP_{15}	0.005	0.001	0.002	0.003	0.003	0.003	0.009	0.010	0.016	0.021	0.016	0.003	<0.001	0.006	0.040	0.016	0.017	*	*	*	*	1.6	-0.1	0.1
VMAT	0.022	0.025	*	*	*	*	*	*	*	*	*	*	*	*	*	*	*	0.029	0.021	0.041	0.030	0.037	1.7	0.3
B-VMAT	<0.001	<0.001	<0.001	<0.001	<0.001	<0.001	<0.001	<0.001	<0.001	0.001	<0.001	<0.001	<0.001	0.003	0.009	0.001	0.004	*	*	*	*	*	<0.001	1.5

Right Breast V4Gy (%)

Treatment B (columns); Treatment A (rows)

	CP_5	CP_6	CP_7	CP_8	CP_9	CP_{10}	CP_{11}	CP_{12}	CP_{13}	CP_{14}	CP_{15}	NCP_5	NCP_6	NCP_7	NCP_8	NCP_9	NCP_{10}	NCP_{11}	NCP_{12}	NCP_{13}	NCP_{14}	NCP_{15}	VMAT	B-VMAT
CP_5	11.8	0.2	0.7	0.9	1.1	1.8	2.3	2.2	2.2	2.4	2.5	1.3	2.9	3.1	3.5	3.7	3.8	3.8	3.7	4.1	4.1	4.2	1.8	4.1
CP_6	0.048	11.0	0.5	0.7	0.9	1.6	2.1	2.0	2.0	2.2	2.3	1.2	2.8	3.0	3.3	3.6	3.7	3.7	3.5	3.9	3.9	4.0	1.6	4.0
CP_7	*	*	11.1	0.2	0.4	1.1	1.7	1.5	1.5	1.7	1.8	0.6	2.3	2.5	2.8	3.1	3.2	3.2	3.0	3.4	3.4	3.5	1.1	3.4
CP_8	0.030	0.044	*	10.8	0.1	0.9	1.4	1.3	1.2	1.4	1.5	0.4	2.1	2.3	2.6	2.9	3.0	3.0	2.8	3.1	3.1	3.3	0.9	3.2
CP_9	*	*	*	*	10.7	0.8	1.3	1.2	1.1	1.3	1.4	0.3	2.0	2.1	2.5	2.7	2.8	2.8	2.7	3.0	3.0	3.2	0.7	3.1
CP_{10}	0.040	0.023	0.004	*	*	9.9	0.5	0.4	0.3	0.5	0.6	-0.5	1.2	1.3	1.7	1.9	2.0	2.0	1.9	2.2	2.2	2.4	-0.0	2.1
CP_{11}	0.004	0.007	0.001	0.008	0.003	0.006	9.4	-0.1	-0.2	0.0	0.1	-1.0	0.6	0.8	1.1	1.4	1.5	1.5	1.4	1.7	1.7	1.9	-0.5	1.6
CP_{12}	0.018	0.011	0.003	0.019	0.020	0.020	*	9.5	-0.1	0.1	0.2	-0.9	0.7	0.9	1.3	1.5	1.6	1.6	1.5	1.8	1.8	2.0	-0.4	1.7
CP_{13}	0.025	0.017	0.010	*	0.028	0.036	*	*	9.6	0.2	0.3	-0.9	0.8	1.0	1.3	1.6	1.7	1.7	1.5	1.9	1.9	2.1	-0.4	1.8
CP_{14}	0.017	0.006	0.002	0.028	0.011	0.002	*	*	*	9.4	0.1	-1.1	0.6	0.7	1.1	1.3	1.5	1.5	1.3	1.7	1.7	1.9	-0.6	1.6
CP_{15}	0.010	0.007	0.002	0.025	0.007	0.012	*	*	*	*	9.3	-1.2	0.4	0.6	1.0	1.2	1.3	1.3	1.2	1.6	1.6	1.8	-0.7	1.4
NCP_5	*	*	*	*	*	*	*	*	*	*	*	10.9	1.7	1.8	2.2	2.4	2.5	2.5	2.4	2.8	2.8	3.0	0.5	2.2
NCP_6	0.049	0.049	0.040	*	*	*	*	*	*	*	*	0.025	9.2	0.2	0.5	0.8	0.9	0.9	0.7	1.1	1.1	1.3	-1.2	0.3
NCP_7	0.024	0.014	0.030	0.040	0.027	*	*	*	*	*	*	0.020	*	9.1	0.4	0.6	0.7	0.7	0.6	1.0	1.0	1.1	-1.3	0.3
NCP_8	0.024	0.010	0.008	0.018	0.013	0.040	*	*	*	*	*	0.033	*	*	8.7	0.2	0.4	0.4	0.2	0.6	0.6	0.8	-1.7	-0.3
NCP_9	0.030	0.016	0.011	0.018	0.011	0.023	*	*	*	*	*	*	*	*	*	8.5	0.1	0.1	-0.0	0.4	0.4	0.5	-2.0	-0.2
NCP_{10}	0.022	0.011	0.014	0.027	0.013	0.022	*	*	*	*	*	*	*	*	*	*	8.4	-0.0	-0.1	0.2	0.2	0.4	-2.1	-0.3
NCP_{11}	0.020	0.007	0.016	0.024	0.020	0.033	*	0.049	*	*	*	*	*	*	*	*	*	8.4	-0.1	0.2	0.2	0.4	-2.1	-0.5
NCP_{12}	0.024	0.011	0.020	0.022	0.021	0.033	*	*	*	*	*	0.046	*	*	*	*	*	*	8.5	0.4	0.4	0.6	-1.9	-0.3
NCP_{13}	0.009	0.006	0.004	0.010	0.009	0.013	*	*	0.032	*	*	0.022	*	*	*	*	*	*	*	7.7	-0.0	0.2	-2.3	-0.7
NCP_{14}	0.005	0.003	0.003	0.011	0.006	0.012	*	*	*	*	*	0.016	*	*	*	*	*	*	*	*	7.7	0.2	-2.3	-0.6
NCP_{15}	0.003	0.002	0.002	0.004	0.005	0.005	0.023	0.019	0.017	0.021	0.033	0.024	*	*	*	*	*	*	*	*	*	7.5	-2.4	-0.7
VMAT	0.023	0.010	0.048	*	*	*	*	*	*	*	*	*	*	*	0.024	0.033	0.022	0.044	*	0.015	0.015	0.015	10.0	2.2
B-VMAT	0.006	0.006	0.008	0.011	0.013	0.022	0.044	*	0.049	*	*	0.031	*	*	*	*	*	*	*	*	*	*	0.039	9.0

Left Breast Dmean (Gy)

Treatment B (columns) / Treatment A (rows)

	CP_{5}	CP_{6}	CP_{7}	CP_{8}	CP_{9}	CP_{10}	CP_{11}	CP_{12}	CP_{13}	CP_{14}	CP_{15}	NCP_{5}	NCP_{6}	NCP_{7}	NCP_{8}	NCP_{9}	NCP_{10}	NCP_{11}	NCP_{12}	NCP_{13}	NCP_{14}	NCP_{15}	VMAT	B-VMAT
CP_{5}	1.9	0.1	0.1	0.1	0.2	0.2	0.2	0.2	0.3	0.3	0.3	0.2	0.3	0.3	0.4	0.4	0.4	0.4	0.4	0.4	0.5	0.5	0.2	0.4
CP_{6}	0.007	1.8	0.0	0.0	0.1	0.1	0.1	0.1	0.1	0.1	0.1	0.1	0.2	0.2	0.3	0.3	0.3	0.3	0.3	0.3	0.3	0.4	0.1	0.3
CP_{7}	*	*	1.8	0.0	0.1	0.1	0.1	0.1	0.1	0.1	0.1	0.1	0.2	0.2	0.3	0.3	0.3	0.3	0.3	0.3	0.3	0.4	0.1	0.3
CP_{8}	0.017	*	0.012	1.8	0.0	0.1	0.1	0.1	0.1	0.1	0.1	0.1	0.1	0.2	0.2	0.3	0.3	0.3	0.3	0.3	0.3	0.3	0.1	0.3
CP_{9}	0.027	*	*	*	1.8	0.1	0.1	0.1	0.1	0.1	0.1	0.0	0.1	0.1	0.2	0.2	0.2	0.3	0.3	0.3	0.3	0.3	0.1	0.3
CP_{10}	0.004	0.011	<0.001	0.002	0.002	1.7	0.0	0.0	0.0	0.0	0.0	-0.0	0.1	0.1	0.2	0.2	0.2	0.2	0.2	0.2	0.2	0.3	0.0	0.2
CP_{11}	0.003	0.002	<0.001	<0.001	0.004	*	1.7	0.0	0.0	0.0	0.0	-0.0	0.0	0.1	0.1	0.2	0.2	0.2	0.2	0.2	0.2	0.2	-0.0	0.2
CP_{12}	0.004	0.002	<0.001	0.002	0.004	*	*	1.7	0.0	0.0	0.0	-0.0	0.0	0.1	0.1	0.1	0.2	0.2	0.2	0.2	0.2	0.2	-0.0	0.2
CP_{13}	0.005	0.007	<0.001	0.004	0.006	*	*	*	1.7	0.0	0.0	-0.0	0.0	0.1	0.1	0.1	0.2	0.2	0.2	0.2	0.2	0.2	-0.0	0.2
CP_{14}	0.003	0.004	<0.001	0.003	0.004	*	*	*	*	1.7	0.0	-0.0	0.0	0.1	0.1	0.1	0.2	0.2	0.2	0.2	0.2	0.2	-0.0	0.2
CP_{15}	0.004	0.004	<0.001	0.010	0.010	*	*	*	*	*	1.7	-0.1	0.0	0.1	0.1	0.1	0.2	0.2	0.2	0.2	0.2	0.2	-0.0	0.2
NCP_{5}	0.042	*	*	*	*	*	*	*	*	*	*	1.7	0.1	0.1	0.2	0.2	0.2	0.2	0.2	0.2	0.3	0.3	0.0	0.2
NCP_{6}	0.004	0.040	0.017	0.029	0.046	*	*	*	*	*	*	*	1.7	0.0	0.1	0.1	0.1	0.1	0.1	0.2	0.2	0.2	-0.1	0.2
NCP_{7}	0.006	0.015	0.013	0.025	0.026	*	*	*	*	*	*	0.036	*	1.6	0.1	0.1	0.1	0.1	0.1	0.1	0.1	0.2	-0.1	0.1
NCP_{8}	0.001	0.002	0.002	0.004	0.004	0.012	0.017	0.046	*	0.047	*	*	*	*	1.6	0.0	0.0	0.0	0.1	0.1	0.1	0.1	-0.2	0.1
NCP_{9}	0.002	0.005	0.004	0.005	0.006	0.014	0.011	0.013	0.021	0.020	0.022	0.033	*	0.047	*	1.6	0.0	0.0	0.0	0.1	0.1	0.1	-0.2	0.0
NCP_{10}	<0.001	0.002	0.002	0.004	0.003	0.006	0.007	0.009	0.007	0.009	0.008	0.039	*	*	*	*	1.5	0.0	0.0	0.0	0.0	0.1	-0.2	0.0
NCP_{11}	0.001	0.003	0.002	0.005	0.004	0.010	0.007	0.010	0.009	0.009	0.013	0.049	*	*	*	*	*	1.5	0.0	0.0	0.0	0.0	-0.2	0.0
NCP_{12}	<0.001	0.002	0.001	0.003	0.003	0.007	0.007	0.011	0.015	0.016	0.019	0.022	0.049	*	*	*	*	0.040	1.5	0.0	0.0	0.0	-0.2	0.0
NCP_{13}	<0.001	0.001	<0.001	<0.001	<0.001	0.002	0.002	0.002	0.002	0.003	0.002	0.040	*	*	*	*	*	*	*	1.5	0.0	0.0	-0.2	-0.0
NCP_{14}	<0.001	<0.001	<0.001	<0.001	<0.001	0.002	0.001	0.002	0.002	0.003	0.003	0.007	0.024	*	*	*	*	*	*	*	1.5	0.0	-0.2	-0.0
NCP_{15}	<0.001	<0.001	<0.001	<0.001	<0.001	0.002	0.003	0.004	0.004	0.004	0.004	0.025	*	*	*	*	*	*	*	*	*	1.5	-0.2	-0.0
VMAT	0.006	*	0.024	*	0.048	*	*	*	*	*	*	*	*	*	0.006	0.010	0.004	0.003	0.003	0.002	0.002	0.001	1.7	0.2
B-VMAT	<0.001	<0.001	<0.001	<0.001	<0.001	<0.001	0.003	0.007	0.006	0.011	0.015	0.016	*	*	*	*	*	*	*	*	*	*	0.002	1.5

Left Breast V4Gy (%)

Treatment B (columns) / Treatment A (rows)

	CP_{5}	CP_{6}	CP_{7}	CP_{8}	CP_{9}	CP_{10}	CP_{11}	CP_{12}	CP_{13}	CP_{14}	CP_{15}	NCP_{5}	NCP_{6}	NCP_{7}	NCP_{8}	NCP_{9}	NCP_{10}	NCP_{11}	NCP_{12}	NCP_{13}	NCP_{14}	NCP_{15}	VMAT	B-VMAT
CP_{5}	12.3	0.1	0.8	1.1	1.1	1.2	1.3	1.5	1.6	1.7	1.5	1.7	1.5	2.2	2.1	2.2	2.5	2.8	2.9	2.7	2.5	2.4	1.5	5.0
CP_{6}	*	12.2	0.7	1.0	1.0	1.1	1.2	1.3	1.4	1.6	1.4	1.6	1.4	2.0	2.0	2.1	2.4	2.6	2.8	2.6	2.3	2.2	1.4	4.8
CP_{7}	*	*	11.5	0.3	0.3	0.4	0.5	0.7	0.8	0.9	0.7	0.9	0.7	1.4	1.3	1.4	1.7	2.0	2.1	1.9	1.7	1.6	0.7	4.2
CP_{8}	*	*	*	11.2	-0.0	0.1	0.2	0.3	0.4	0.6	0.4	0.6	0.4	1.0	1.0	1.1	1.4	1.6	1.8	1.6	1.4	1.2	0.4	3.8
CP_{9}	*	*	*	*	11.2	0.1	0.2	0.4	0.5	0.6	0.4	0.6	0.4	1.0	1.0	1.1	1.4	1.7	1.8	1.6	1.4	1.3	0.4	3.9
CP_{10}	*	*	*	*	*	11.1	0.1	0.3	0.4	0.5	0.3	0.6	0.4	1.0	1.0	1.1	1.3	1.6	1.7	1.5	1.3	1.2	0.3	3.8
CP_{11}	*	*	*	*	*	*	11.0	0.1	0.2	0.4	0.2	0.4	0.2	0.8	0.8	0.9	1.2	1.4	1.6	1.4	1.2	1.0	0.2	3.6
CP_{12}	0.040	*	*	*	*	*	*	10.8	0.1	0.2	0.0	0.3	0.1	0.7	0.7	0.8	1.0	1.3	1.4	1.2	1.0	0.9	0.1	3.5
CP_{13}	0.048	*	*	*	*	*	*	*	10.7	0.1	-0.1	0.2	-0.0	0.6	0.6	0.7	0.9	1.2	1.3	1.1	0.9	0.8	-0.0	3.4
CP_{14}	0.017	0.025	0.046	*	*	*	0.048	*	*	10.6	-0.2	0.0	-0.2	0.5	0.4	0.5	0.8	1.1	1.2	1.0	0.8	0.7	-0.2	3.3
CP_{15}	0.023	0.023	*	*	*	*	*	*	*	*	10.8	0.3	0.0	0.7	0.6	0.7	1.0	1.3	1.4	1.2	1.0	0.9	0.0	3.5
NCP_{5}	*	*	*	*	*	*	*	*	*	*	*	10.5	-0.2	0.4	0.4	0.5	0.8	1.0	1.2	0.9	0.7	0.6	-0.2	3.2
NCP_{6}	*	*	*	*	*	*	*	*	*	*	*	*	10.8	0.6	0.6	0.7	1.0	1.2	1.4	1.2	0.9	0.8	-0.0	3.4
NCP_{7}	0.048	*	*	*	*	*	*	*	*	*	*	*	*	10.1	-0.0	0.1	0.4	0.6	0.8	0.5	0.3	0.2	-0.6	2.8
NCP_{8}	0.040	*	0.037	*	*	*	*	*	*	*	*	*	*	*	10.2	0.1	0.4	0.6	0.8	0.6	0.3	0.2	-0.6	2.8
NCP_{9}	0.030	*	*	*	*	*	*	*	*	*	*	*	*	*	*	10.1	0.3	0.5	0.7	0.5	0.2	0.1	-0.7	2.7
NCP_{10}	0.025	0.037	0.040	*	*	*	*	*	*	*	*	*	*	*	*	*	9.8	0.3	0.4	0.2	-0.0	-0.1	-1.0	2.5
NCP_{11}	0.012	0.030	0.021	*	0.048	*	*	*	*	*	*	*	*	*	*	*	0.010	9.5	0.1	-0.1	-0.3	-0.4	-1.2	2.2
NCP_{12}	0.011	0.023	0.028	*	0.048	0.040	0.050	*	*	*	*	*	*	*	*	*	*	*	9.4	-0.2	-0.4	-0.5	-1.4	2.1
NCP_{13}	0.015	0.023	0.040	*	*	*	*	*	*	*	*	*	*	*	*	*	*	*	*	9.6	-0.2	-0.3	-1.2	2.3
NCP_{14}	0.025	0.044	*	*	*	*	*	*	*	*	*	*	*	*	*	*	*	*	*	*	9.8	-0.1	-1.0	2.5
NCP_{15}	0.026	0.044	*	*	*	*	*	*	*	*	*	*	*	*	*	*	*	*	0.030	*	*	9.9	-0.8	2.6
VMAT	0.019	0.024	*	*	*	*	*	*	*	*	*	*	*	*	*	*	*	*	*	*	*	*	10.8	3.4
B-VMAT	<0.001	<0.001	<0.001	<0.001	<0.001	<0.001	<0.001	0.001	0.002	0.002	0.002	0.004	0.002	0.002	0.001	<0.001	0.002	0.004	0.006	0.005	0.005	0.004	<0.001	7.3

Heart Dmean (Gy)

Treatment B (columns) / **Treatment A** (rows)

	CP_5	CP_6	CP_7	CP_8	CP_9	CP_{10}	CP_{11}	CP_{12}	CP_{13}	CP_{14}	CP_{15}	NCP_5	NCP_6	NCP_7	NCP_8	NCP_9	NCP_{10}	NCP_{11}	NCP_{12}	NCP_{13}	NCP_{14}	NCP_{15}	VMAT	B-VMAT
CP_5	6.6	0.2	0.4	0.6	0.7	0.8	0.8	0.9	0.9	0.9	1.0	0.8	0.9	1.0	1.1	1.3	1.3	1.4	1.5	1.5	1.6	1.6	0.9	1.2
CP_6	<0.001	6.4	0.2	0.4	0.5	0.6	0.6	0.7	0.7	0.7	0.8	0.6	0.7	0.9	0.9	1.1	1.1	1.2	1.3	1.3	1.4	1.4	0.7	1.0
CP_7	<0.001	<0.001	6.2	0.2	0.3	0.4	0.5	0.5	0.5	0.6	0.6	0.4	0.5	0.7	0.7	0.9	1.0	1.0	1.1	1.2	1.2	1.3	0.6	0.8
CP_8	<0.001	<0.001	<0.001	6.1	0.2	0.2	0.3	0.3	0.3	0.4	0.4	0.2	0.3	0.5	0.5	0.7	0.8	0.8	0.9	1.0	1.0	1.1	0.4	0.6
CP_9	<0.001	<0.001	<0.001	0.001	5.9	0.0	0.1	0.2	0.2	0.2	0.2	0.0	0.2	0.3	0.4	0.6	0.6	0.7	0.7	0.8	0.9	0.9	0.2	0.5
CP_{10}	<0.001	<0.001	<0.001	<0.001	0.002	5.9	0.1	0.1	0.1	0.2	0.2	-0.0	0.1	0.3	0.3	0.5	0.6	0.6	0.7	0.8	0.8	0.9	0.2	0.4
CP_{11}	<0.001	<0.001	<0.001	<0.001	<0.001	<0.001	5.8	0.1	0.1	0.1	0.1	-0.1	0.0	0.2	0.3	0.4	0.5	0.6	0.6	0.7	0.7	0.8	0.1	0.4
CP_{12}	<0.001	<0.001	<0.001	<0.001	<0.001	<0.001	0.001	5.7	-0.0	0.0	0.1	-0.2	-0.0	0.1	0.2	0.4	0.4	0.5	0.6	0.6	0.7	0.7	0.0	0.3
CP_{13}	<0.001	<0.001	<0.001	<0.001	<0.001	<0.001	0.004	0.039	5.7	0.0	0.1	-0.1	-0.0	0.1	0.2	0.4	0.4	0.5	0.6	0.6	0.7	0.7	0.0	0.3
CP_{14}	<0.001	<0.001	<0.001	<0.001	<0.001	<0.001	<0.001	0.023	0.003	5.7	0.0	-0.2	-0.1	0.1	0.2	0.3	0.4	0.4	0.5	0.6	0.6	0.7	-0.0	0.3
CP_{15}	<0.001	<0.001	<0.001	<0.001	<0.001	<0.001	<0.001	0.007	<0.001	0.005	5.7	-0.2	-0.1	0.1	0.1	0.3	0.4	0.4	0.5	0.6	0.6	0.7	-0.0	0.2
NCP_5	<0.001	<0.001	0.004	*	*	*	*	*	*	*	*	5.9	0.1	0.3	0.3	0.5	0.6	0.6	0.7	0.8	0.8	0.9	0.2	0.5
NCP_6	<0.001	<0.001	<0.001	0.007	0.034	*	*	*	*	*	*	0.011	5.7	0.2	0.2	0.4	0.4	0.5	0.6	0.6	0.7	0.7	0.0	0.3
NCP_7	<0.001	<0.001	<0.001	0.002	0.006	0.009	0.041	*	*	*	*	<0.001	<0.001	5.6	0.1	0.2	0.3	0.3	0.4	0.5	0.5	0.6	-0.1	0.2
NCP_8	<0.001	<0.001	<0.001	<0.001	<0.001	0.002	0.007	0.046	0.036	0.048	*	<0.001	<0.001	0.003	5.5	0.2	0.2	0.3	0.4	0.4	0.5	0.5	-0.2	0.1
NCP_9	<0.001	<0.001	<0.001	<0.001	<0.001	<0.001	<0.001	<0.001	<0.001	<0.001	0.001	<0.001	<0.001	<0.001	<0.001	5.4	0.1	0.1	0.2	0.3	0.3	0.4	-0.3	-0.1
NCP_{10}	<0.001	<0.001	<0.001	<0.001	<0.001	<0.001	<0.001	<0.001	<0.001	<0.001	0.001	<0.001	<0.001	<0.001	<0.001	0.002	5.3	0.1	0.1	0.2	0.3	0.3	-0.4	-0.1
NCP_{11}	<0.001	<0.001	<0.001	<0.001	<0.001	<0.001	<0.001	0.001	<0.001	<0.001	<0.001	<0.001	<0.001	<0.001	<0.001	0.002	<0.001	5.2	0.1	0.1	0.2	0.2	-0.5	-0.2
NCP_{12}	<0.001	<0.001	<0.001	<0.001	<0.001	<0.001	<0.001	<0.001	<0.001	<0.001	0.001	<0.001	<0.001	<0.001	<0.001	0.002	<0.001	0.003	5.2	0.1	0.1	0.2	-0.5	-0.2
NCP_{13}	<0.001	<0.001	<0.001	<0.001	<0.001	<0.001	<0.001	<0.001	<0.001	<0.001	<0.001	<0.001	<0.001	<0.001	<0.001	0.001	<0.001	<0.001	<0.001	5.1	0.1	0.1	-0.6	-0.3
NCP_{14}	<0.001	<0.001	<0.001	<0.001	<0.001	<0.001	<0.001	<0.001	<0.001	<0.001	<0.001	<0.001	<0.001	<0.001	<0.001	<0.001	<0.001	<0.001	<0.001	<0.001	5.0	0.0	-0.7	-0.4
NCP_{15}	<0.001	<0.001	<0.001	<0.001	<0.001	<0.001	<0.001	<0.001	<0.001	<0.001	<0.001	<0.001	<0.001	<0.001	<0.001	<0.001	<0.001	<0.001	<0.001	<0.001	0.001	5.0	-0.7	-0.4
VMAT	<0.001	<0.001	<0.001	<0.001	0.004	0.020	*	*	*	*	*	*	*	*	*	0.001	<0.001	<0.001	<0.001	<0.001	<0.001	<0.001	5.7	0.3
B-VMAT	<0.001	<0.001	<0.001	<0.001	<0.001	0.001	0.002	0.004	0.002	0.003	0.005	0.004	0.017	*	*	*	*	*	0.036	0.009	0.003	0.002	0.004	5.4

Lungs Dmean (Gy)

Treatment B (columns) / **Treatment A** (rows)

	CP_5	CP_6	CP_7	CP_8	CP_9	CP_{10}	CP_{11}	CP_{12}	CP_{13}	CP_{14}	CP_{15}	NCP_5	NCP_6	NCP_7	NCP_8	NCP_9	NCP_{10}	NCP_{11}	NCP_{12}	NCP_{13}	NCP_{14}	NCP_{15}	VMAT	B-VMAT
CP_5	8.6	0.3	0.5	0.7	0.8	0.9	1.0	1.1	1.1	1.2	1.2	0.4	0.7	0.9	1.2	1.3	1.4	1.5	1.5	1.6	1.7	1.7	1.1	1.4
CP_6	<0.001	8.3	0.2	0.4	0.5	0.6	0.7	0.7	0.8	0.9	0.9	0.1	0.4	0.6	0.9	1.0	1.1	1.2	1.2	1.3	1.3	1.4	0.8	1.1
CP_7	<0.001	<0.001	8.1	0.2	0.3	0.4	0.4	0.5	0.6	0.6	0.7	-0.1	0.2	0.4	0.6	0.8	0.8	0.9	1.0	1.1	1.1	1.2	0.6	0.9
CP_8	<0.001	<0.001	<0.001	7.9	0.1	0.2	0.3	0.4	0.4	0.5	0.5	-0.3	0.0	0.2	0.5	0.6	0.7	0.8	0.8	0.9	1.0	1.0	0.4	0.7
CP_9	<0.001	<0.001	<0.001	<0.001	7.8	0.1	0.2	0.2	0.3	0.4	0.4	-0.4	-0.1	0.1	0.4	0.5	0.6	0.7	0.7	0.8	0.9	0.9	0.3	0.6
CP_{10}	<0.001	<0.001	<0.001	<0.001	<0.001	7.7	0.1	0.1	0.2	0.3	0.3	-0.5	-0.2	0.0	0.3	0.4	0.5	0.6	0.6	0.7	0.8	0.8	0.2	0.5
CP_{11}	<0.001	<0.001	<0.001	<0.001	<0.001	<0.001	7.6	0.1	0.1	0.2	0.2	-0.6	-0.3	-0.0	0.2	0.3	0.4	0.5	0.6	0.6	0.7	0.7	0.2	0.4
CP_{12}	<0.001	<0.001	<0.001	<0.001	<0.001	<0.001	<0.001	7.5	0.1	0.1	0.2	-0.6	-0.3	-0.1	0.1	0.2	0.3	0.4	0.5	0.6	0.6	0.7	0.1	0.4
CP_{13}	<0.001	<0.001	<0.001	<0.001	<0.001	<0.001	<0.001	<0.001	7.5	0.1	0.1	-0.7	-0.4	-0.2	0.1	0.2	0.3	0.4	0.4	0.5	0.5	0.6	0.0	0.3
CP_{14}	<0.001	<0.001	<0.001	<0.001	<0.001	<0.001	<0.001	<0.001	<0.001	7.4	0.0	-0.8	-0.5	-0.2	0.0	0.1	0.2	0.3	0.4	0.4	0.5	0.5	-0.0	0.2
CP_{15}	<0.001	<0.001	<0.001	<0.001	<0.001	<0.001	<0.001	<0.001	0.001	0.019	7.4	-0.8	-0.5	-0.3	-0.0	0.1	0.2	0.3	0.3	0.4	0.5	0.5	-0.1	0.2
NCP_5	0.014	*	*	0.040	0.013	0.003	0.001	<0.001	<0.001	<0.001	<0.001	8.2	0.3	0.5	0.8	0.9	1.0	1.1	1.1	1.2	1.3	1.3	0.7	1.0
NCP_6	<0.001	0.001	*	*	*	*	*	0.011	0.004	0.001	<0.001	<0.001	7.9	0.2	0.5	0.6	0.7	0.8	0.8	0.9	0.9	1.0	0.4	0.7
NCP_7	<0.001	<0.001	0.002	0.034	*	*	*	*	*	0.020	0.010	<0.001	<0.001	7.7	0.2	0.4	0.4	0.5	0.6	0.7	0.7	0.8	0.2	0.5
NCP_8	<0.001	<0.001	<0.001	<0.001	0.004	*	*	*	*	*	*	<0.001	<0.001	<0.001	7.4	0.1	0.2	0.3	0.4	0.4	0.5	0.5	-0.1	0.2
NCP_9	<0.001	<0.001	<0.001	<0.001	<0.001	<0.001	0.005	*	*	*	*	<0.001	<0.001	<0.001	<0.001	7.3	0.1	0.2	0.2	0.3	0.4	0.4	-0.2	0.1
NCP_{10}	<0.001	<0.001	<0.001	<0.001	<0.001	<0.001	<0.001	0.005	0.048	*	*	<0.001	<0.001	<0.001	<0.001	<0.001	7.2	0.1	0.2	0.2	0.3	0.3	-0.3	0.0
NCP_{11}	<0.001	<0.001	<0.001	<0.001	<0.001	<0.001	<0.001	<0.001	0.005	0.011	0.021	<0.001	<0.001	<0.001	<0.001	<0.001	<0.001	7.1	0.1	0.1	0.2	0.2	-0.4	-0.1
NCP_{12}	<0.001	<0.001	<0.001	<0.001	<0.001	<0.001	<0.001	<0.001	0.001	0.002	0.003	<0.001	<0.001	<0.001	<0.001	<0.001	<0.001	0.013	7.1	0.1	0.1	0.2	-0.4	-0.1
NCP_{13}	<0.001	<0.001	<0.001	<0.001	<0.001	<0.001	<0.001	<0.001	<0.001	<0.001	<0.001	<0.001	<0.001	<0.001	<0.001	<0.001	<0.001	<0.001	<0.001	7.0	0.0	0.1	-0.5	-0.2
NCP_{14}	<0.001	<0.001	<0.001	<0.001	<0.001	<0.001	<0.001	<0.001	<0.001	<0.001	<0.001	<0.001	<0.001	<0.001	<0.001	<0.001	<0.001	<0.001	<0.001	<0.001	6.9	0.1	-0.5	-0.2
NCP_{15}	<0.001	<0.001	<0.001	<0.001	<0.001	<0.001	<0.001	<0.001	<0.001	<0.001	<0.001	<0.001	<0.001	<0.001	<0.001	<0.001	<0.001	<0.001	<0.001	<0.001	<0.001	6.9	-0.6	-0.3
VMAT	<0.001	<0.001	<0.001	<0.001	<0.001	0.003	0.013	*	*	*	0.033	<0.001	0.008	*	*	*	0.025	0.002	<0.001	<0.001	<0.001	<0.001	7.5	0.3
B-VMAT	<0.001	<0.001	<0.001	<0.001	<0.001	<0.001	0.002	0.011	0.023	*	*	<0.001	<0.001	<0.001	0.020	*	*	*	*	*	0.037	0.013	0.041	7.2

Lungs V5Gy (%)

Treatment B (columns) / Treatment A (rows)

Treatment A \ Treatment B	CP_5	CP_6	CP_7	CP_8	CP_9	CP_{10}	CP_{11}	CP_{12}	CP_{13}	CP_{14}	CP_{15}	NCP_5	NCP_6	NCP_7	NCP_8	NCP_9	NCP_{10}	NCP_{11}	NCP_{12}	NCP_{13}	NCP_{14}	NCP_{15}	VMAT	B-VMAT
CP_5	44.3	1.1	1.9	2.6	3.0	3.7	4.1	4.4	4.7	4.9	4.7	4.0	5.0	6.0	7.0	7.3	7.8	8.1	8.3	8.6	8.7	8.8	3.4	11.0
CP_6	*	43.3	0.9	1.5	1.9	2.6	3.0	3.4	3.6	3.8	3.7	3.0	4.0	5.0	5.9	6.3	6.8	7.1	7.3	7.6	7.6	7.8	2.4	10.0
CP_7	0.014	0.042	42.4	0.7	1.1	1.8	2.2	2.5	2.8	3.0	2.8	2.1	3.1	4.1	5.1	5.4	5.9	6.2	6.4	6.7	6.8	6.9	1.5	9.1
CP_8	0.002	<0.001	0.004	41.7	0.4	1.1	1.5	1.8	2.1	2.3	2.2	1.4	2.4	3.5	4.4	4.8	5.2	5.5	5.7	6.0	6.1	6.2	0.8	8.4
CP_9	0.002	<0.001	0.007	*	41.4	0.7	1.1	1.4	1.7	1.9	1.8	1.0	2.0	3.1	4.0	4.4	4.9	5.2	5.4	5.7	5.7	5.9	0.4	8.0
CP_{10}	<0.001	<0.001	0.001	0.008	0.021	40.6	0.4	0.7	1.0	1.2	1.1	0.3	1.3	2.4	3.3	3.7	4.2	4.4	4.7	5.0	5.0	5.2	-0.3	7.3
CP_{11}	<0.001	<0.001	<0.001	0.002	0.002	0.034	40.2	0.3	0.6	0.8	0.7	-0.1	0.9	2.0	2.9	3.3	3.8	4.0	4.3	4.6	4.6	4.8	-0.7	6.9
CP_{12}	<0.001	<0.001	<0.001	<0.001	<0.001	0.001	0.019	39.9	0.3	0.5	0.3	-0.4	0.6	1.6	2.6	2.9	3.4	3.7	3.9	4.2	4.3	4.4	-1.0	6.6
CP_{13}	<0.001	<0.001	<0.001	<0.001	<0.001	0.002	0.011	*	39.6	0.2	0.1	-0.7	0.3	1.4	2.3	2.6	3.1	3.4	3.6	3.9	4.0	4.1	-1.3	6.3
CP_{14}	<0.001	<0.001	<0.001	<0.001	<0.001	0.002	0.005	*	*	39.4	-0.1	-0.9	0.1	1.2	2.1	2.5	3.0	3.3	3.5	3.8	3.8	4.0	-1.5	6.1
CP_{15}	<0.001	<0.001	<0.001	<0.001	<0.001	0.009	0.016	*	*	*	39.6	-0.7	0.3	1.3	2.2	2.6	3.1	3.4	3.6	3.9	3.9	4.1	-1.3	6.3
NCP_5	<0.001	0.005	*	*	*	*	*	*	*	*	*	40.3	1.0	2.0	3.0	3.3	3.8	4.1	4.3	4.6	4.7	4.8	-0.6	7.0
NCP_6	<0.001	<0.001	0.012	0.030	*	*	*	*	*	*	*	0.005	39.3	1.0	2.0	2.3	2.8	3.1	3.3	3.6	3.7	3.8	-1.6	6.0
NCP_7	<0.001	<0.001	0.003	0.007	0.015	0.044	*	*	*	*	*	0.005	0.008	38.3	0.9	1.3	1.8	2.1	2.3	2.6	2.6	2.8	-2.6	5.0
NCP_8	<0.001	<0.001	<0.001	0.002	0.005	0.017	0.040	*	*	*	*	<0.001	<0.001	<0.001	37.3	0.4	0.9	1.1	1.4	1.7	1.7	1.9	-3.6	4.0
NCP_9	<0.001	<0.001	<0.001	<0.001	0.002	0.011	0.021	0.034	*	*	0.042	<0.001	<0.001	<0.001	*	37.0	0.5	0.8	1.0	1.3	1.3	1.5	-3.9	3.7
NCP_{10}	<0.001	<0.001	<0.001	<0.001	0.001	0.004	0.009	0.013	0.015	0.019	0.017	<0.001	<0.001	<0.001	*	*	36.5	0.3	0.5	0.8	0.8	1.0	-4.4	3.2
NCP_{11}	<0.001	<0.001	<0.001	<0.001	<0.001	0.003	0.007	0.010	0.011	0.010	0.009	<0.001	<0.001	<0.001	*	*	*	36.2	0.2	0.5	0.6	0.7	-4.7	2.9
NCP_{12}	<0.001	<0.001	<0.001	<0.001	<0.001	0.002	0.004	0.005	0.010	0.008	0.007	<0.001	<0.001	<0.001	0.035	*	*	*	36.0	0.3	0.3	0.5	-4.9	2.7
NCP_{13}	<0.001	<0.001	<0.001	<0.001	<0.001	0.001	0.003	0.003	0.005	0.004	0.004	<0.001	<0.001	<0.001	0.014	0.028	0.042	*	*	35.7	0.0	0.2	-5.2	2.4
NCP_{14}	<0.001	<0.001	<0.001	<0.001	<0.001	<0.001	0.002	0.002	0.003	0.002	0.002	<0.001	<0.001	<0.001	0.017	0.014	0.028	*	*	*	35.6	0.2	-5.3	2.3
NCP_{15}	<0.001	<0.001	<0.001	<0.001	<0.001	<0.001	0.002	0.002	0.003	0.002	0.002	<0.001	<0.001	<0.001	0.008	0.013	0.017	*	*	*	*	35.5	-5.4	2.2
VMAT	0.002	0.006	*	*	*	*	*	0.017	0.003	<0.001	0.001	*	*	0.012	0.002	<0.001	<0.001	<0.001	<0.001	<0.001	<0.001	<0.001	40.9	7.6
B-VMAT	<0.001	<0.001	<0.001	<0.001	<0.001	<0.001	<0.001	<0.001	<0.001	<0.001	<0.001	<0.001	<0.001	<0.001	<0.001	<0.001	<0.001	0.001	<0.001	0.001	0.002	0.003	<0.001	33.3

Lungs V20Gy (%)

Treatment B (columns) / Treatment A (rows)

Treatment A \ Treatment B	CP_5	CP_6	CP_7	CP_8	CP_9	CP_{10}	CP_{11}	CP_{12}	CP_{13}	CP_{14}	CP_{15}	NCP_5	NCP_6	NCP_7	NCP_8	NCP_9	NCP_{10}	NCP_{11}	NCP_{12}	NCP_{13}	NCP_{14}	NCP_{15}	VMAT	B-VMAT
CP_5	17.4	1.3	2.1	2.5	3.0	3.2	3.4	3.6	3.8	4.0	4.2	-0.2	1.2	1.9	2.8	3.3	3.6	4.0	4.3	4.5	4.7	5.0	4.4	1.5
CP_6	<0.001	16.1	0.8	1.2	1.7	1.9	2.1	2.3	2.5	2.7	2.9	-1.5	-0.1	0.6	1.5	2.0	2.3	2.7	2.9	3.2	3.4	3.6	3.1	0.2
CP_7	<0.001	<0.001	15.3	0.4	0.9	1.1	1.3	1.5	1.7	1.9	2.1	-2.3	-0.9	-0.2	0.7	1.2	1.5	1.8	2.1	2.4	2.6	2.8	2.3	-0.6
CP_8	<0.001	<0.001	<0.001	14.8	0.5	0.7	0.9	1.0	1.3	1.5	1.7	-2.8	-1.3	-0.6	0.3	0.8	1.1	1.4	1.7	1.9	2.2	2.4	1.8	-1.0
CP_9	<0.001	<0.001	<0.001	<0.001	14.4	0.2	0.4	0.6	0.8	1.0	1.2	-3.2	-1.8	-1.1	-0.1	0.3	0.6	1.0	1.3	1.5	1.7	2.0	1.4	-1.5
CP_{10}	<0.001	<0.001	<0.001	<0.001	0.042	14.2	0.2	0.4	0.6	0.8	1.0	-3.4	-2.0	-1.3	-0.4	0.1	0.4	0.8	1.0	1.3	1.5	1.7	1.2	-1.7
CP_{11}	<0.001	<0.001	<0.001	<0.001	0.015	0.014	14.0	0.2	0.4	0.6	0.8	-3.6	-2.2	-1.5	-0.6	-0.1	0.2	0.6	0.9	1.1	1.3	1.6	1.0	-1.9
CP_{12}	<0.001	<0.001	<0.001	<0.001	0.001	0.001	0.003	13.8	0.2	0.4	0.6	-3.8	-2.4	-1.7	-0.7	-0.3	0.0	0.4	0.7	0.9	1.1	1.4	0.8	-2.1
CP_{13}	<0.001	<0.001	<0.001	<0.001	<0.001	<0.001	<0.001	<0.001	13.6	0.2	0.4	-4.0	-2.6	-1.9	-1.0	-0.5	-0.2	0.2	0.4	0.7	0.9	1.1	0.6	-2.3
CP_{14}	<0.001	<0.001	<0.001	<0.001	<0.001	<0.001	<0.001	<0.001	<0.001	13.4	0.2	-4.2	-2.8	-2.1	-1.2	-0.7	-0.4	-0.0	0.2	0.5	0.7	1.0	0.4	-2.5
CP_{15}	<0.001	<0.001	<0.001	<0.001	<0.001	<0.001	<0.001	<0.001	<0.001	0.002	13.2	-4.4	-3.0	-2.3	-1.4	-0.9	-0.6	-0.3	0.0	0.2	0.5	0.7	0.2	-2.7
NCP_5	*	<0.001	<0.001	<0.001	<0.001	<0.001	<0.001	<0.001	<0.001	<0.001	<0.001	17.6	1.4	2.1	3.1	3.5	3.8	4.2	4.5	4.7	4.9	5.2	4.6	1.7
NCP_6	0.001	*	0.001	<0.001	<0.001	<0.001	<0.001	<0.001	<0.001	<0.001	<0.001	<0.001	16.2	0.7	1.6	2.1	2.4	2.8	3.0	3.3	3.5	3.7	3.2	0.3
NCP_7	<0.001	*	*	0.003	<0.001	<0.001	<0.001	<0.001	<0.001	<0.001	<0.001	<0.001	<0.001	15.5	0.9	1.4	1.7	2.0	2.3	2.6	2.8	3.0	2.5	-0.4
NCP_8	<0.001	<0.001	<0.001	*	*	0.017	0.002	<0.001	<0.001	<0.001	<0.001	<0.001	<0.001	<0.001	14.5	0.5	0.7	1.1	1.4	1.6	1.9	2.1	1.5	-1.3
NCP_9	<0.001	<0.001	<0.001	<0.001	0.048	*	*	0.040	0.006	0.002	<0.001	<0.001	<0.001	<0.001	<0.001	14.1	0.3	0.6	0.9	1.2	1.4	1.6	1.1	-1.8
NCP_{10}	<0.001	<0.001	<0.001	<0.001	0.011	*	*	*	*	*	0.007	<0.001	<0.001	<0.001	<0.001	<0.001	13.8	0.4	0.7	0.9	1.1	1.4	0.8	-2.1
NCP_{11}	<0.001	<0.001	<0.001	<0.001	<0.001	<0.001	*	*	*	*	*	<0.001	<0.001	<0.001	<0.001	<0.001	<0.001	13.4	0.3	0.5	0.8	1.0	0.4	-2.5
NCP_{12}	<0.001	<0.001	<0.001	<0.001	<0.001	<0.001	<0.001	0.007	*	*	*	<0.001	<0.001	<0.001	<0.001	<0.001	<0.001	<0.001	13.1	0.2	0.5	0.7	0.1	-2.7
NCP_{13}	<0.001	<0.001	<0.001	<0.001	<0.001	<0.001	<0.001	<0.001	0.013	*	*	<0.001	<0.001	<0.001	<0.001	<0.001	<0.001	<0.001	<0.001	12.9	0.2	0.5	-0.1	-3.0
NCP_{14}	<0.001	<0.001	<0.001	<0.001	<0.001	<0.001	<0.001	<0.001	<0.001	0.004	0.030	<0.001	<0.001	<0.001	<0.001	<0.001	<0.001	<0.001	<0.001	<0.001	12.7	0.2	-0.3	-3.2
NCP_{15}	<0.001	<0.001	<0.001	<0.001	<0.001	<0.001	<0.001	<0.001	<0.001	<0.001	0.002	<0.001	<0.001	<0.001	<0.001	<0.001	<0.001	<0.001	<0.001	<0.001	<0.001	12.4	-0.6	-3.4
VMAT	<0.001	<0.001	<0.001	<0.001	<0.001	<0.001	<0.001	0.001	0.006	0.016	*	<0.001	<0.001	<0.001	<0.001	<0.001	0.003	*	*	*	0.048	0.005	13.0	-2.9
B-VMAT	0.007	*	*	0.017	0.002	<0.001	<0.001	<0.001	<0.001	<0.001	<0.001	<0.001	*	*	<0.001	<0.001	<0.001	<0.001	<0.001	<0.001	<0.001	<0.001	<0.001	15.9

Patient V5Gy (cc)

Treatment B (columns) / Treatment A (rows)

	CP5	CP6	CP7	CP8	CP9	CP10	CP11	CP12	CP13	CP14	CP15	NCP5	NCP6	NCP7	NCP8	NCP9	NCP10	NCP11	NCP12	NCP13	NCP14	NCP15	VMAT	B-VMAT
CP5	5246.	70.0	185.6	265.3	292.3	319.7	339.4	355.2	373.4	386.4	362.3	271.8	298.4	384.5	420.6	452.1	497.6	530.3	539.3	556.4	560.0	581.9	170.8	1055.4
CP6	*	5176.	115.7	195.3	222.3	249.8	269.5	285.2	303.5	316.5	292.4	201.8	228.4	314.6	350.7	382.2	427.6	460.4	469.3	486.4	490.1	511.9	100.8	985.4
CP7	0.006	0.005	5061.	79.6	106.6	134.1	153.8	169.5	187.8	200.8	176.7	86.1	112.7	198.9	235.0	266.5	311.9	344.7	353.6	370.8	374.4	396.2	-14.9	869.8
CP8	0.001	<0.001	0.007	4981.	27.0	54.4	74.2	89.9	108.2	121.1	97.1	6.5	33.1	119.2	155.3	186.8	232.3	265.1	274.0	291.1	294.7	316.6	-94.5	790.1
CP9	0.001	<0.001	0.009	*	4954.	27.4	47.2	62.9	81.1	94.1	70.1	-20.5	6.1	92.2	128.3	159.8	205.3	238.0	247.0	264.1	267.7	289.6	121.5	763.1
CP10	<0.001	<0.001	0.003	*	*	4927.	19.7	35.5	53.7	66.7	42.6	-48.0	-21.3	64.8	100.9	132.4	177.9	210.6	219.6	236.7	240.3	262.2	148.9	735.7
CP11	<0.001	<0.001	<0.001	0.015	*	0.026	4907.	15.7	34.0	47.0	22.9	-67.7	-41.1	45.1	81.2	112.7	158.1	190.9	199.8	216.9	220.6	242.4	168.7	716.0
CP12	<0.001	<0.001	<0.001	0.007	*	*	*	4891.	18.3	31.3	7.2	-83.4	-56.8	29.4	65.5	97.0	142.4	175.2	184.1	201.2	204.8	226.7	184.4	700.2
CP13	<0.001	<0.001	0.001	0.001	0.025	0.013	*	*	4873.	13.0	-11.1	101.7	-75.1	11.1	47.2	78.7	124.1	156.9	165.8	183.0	186.6	208.5	202.6	682.0
CP14	<0.001	<0.001	0.001	0.002	0.013	0.015	*	*	*	4860.	-24.1	114.7	-88.0	-1.9	34.2	65.7	111.1	143.9	152.9	170.0	173.6	195.5	215.6	669.0
CP15	<0.001	<0.001	<0.001	0.005	0.032	*	*	*	*	*	4884.	-90.6	-64.0	22.2	58.3	89.8	135.2	168.0	176.9	194.1	197.7	219.5	191.6	693.1
NCP5	0.001	0.012	*	*	*	*	*	*	*	*	*	4975.	26.6	112.8	148.9	180.4	225.8	258.6	267.5	284.6	288.2	310.1	101.0	783.6
NCP6	0.001	0.017	*	*	*	*	*	*	*	*	*	*	4948.	86.1	122.2	153.7	199.2	232.0	240.9	258.0	261.6	283.5	127.6	757.0
NCP7	<0.001	0.006	*	*	*	*	*	*	*	*	*	0.013	0.017	4862.	36.1	67.6	113.1	145.8	154.8	171.9	175.5	197.4	213.7	670.9
NCP8	<0.001	<0.001	0.015	*	*	*	*	*	*	*	*	0.007	0.002	*	4826.	31.5	76.9	109.7	118.7	135.8	139.4	161.3	249.8	634.8
NCP9	<0.001	<0.001	0.004	0.048	*	*	*	*	*	*	*	0.003	0.001	0.014	*	4794.	45.4	78.2	87.2	104.3	107.9	129.8	281.3	603.3
NCP10	<0.001	<0.001	0.003	0.025	*	*	*	*	*	*	*	<0.001	<0.001	0.003	*	*	4749.	32.8	41.7	58.8	62.4	84.3	-326.8	557.8
NCP11	<0.001	<0.001	0.002	0.009	0.025	0.026	0.026	0.028	*	*	0.042	<0.001	<0.001	0.001	0.035	*	0.004	4716.	8.9	26.1	29.7	51.6	-359.6	525.1
NCP12	<0.001	<0.001	0.001	0.004	0.012	0.014	0.030	0.026	0.045	*	0.025	<0.001	<0.001	0.001	0.019	0.026	0.037	*	4707.	17.1	20.7	42.6	-368.5	516.1
NCP13	<0.001	<0.001	<0.001	0.005	0.016	0.013	0.023	0.021	*	*	0.023	<0.001	<0.001	<0.001	0.006	0.005	0.009	*	*	4690.	3.6	25.5	-385.6	499.0
NCP14	<0.001	<0.001	<0.001	0.003	0.007	0.006	0.009	0.012	0.025	0.048	0.013	<0.001	<0.001	<0.001	0.004	0.003	0.010	*	*	*	4686.	21.9	-389.2	495.4
NCP15	<0.001	<0.001	<0.001	<0.001	0.002	0.003	0.006	0.005	0.009	0.009	0.006	<0.001	<0.001	<0.001	0.002	<0.001	0.003	0.021	0.028	*	*	4665.	-411.1	473.5
VMAT	*	*	*	0.040	0.011	0.012	0.001	<0.001	<0.001	<0.001	<0.001	*	*	0.007	0.002	<0.001	<0.001	<0.001	<0.001	<0.001	<0.001	<0.00	5076.	884.6
B-VMAT	<0.001	<0.001	<0.001	<0.001	<0.001	<0.001	<0.001	<0.001	<0.001	<0.001	<0.001	<0.001	<0.001	<0.001	<0.001	<0.001	<0.001	<0.001	<0.001	<0.001	<0.001	<0.001	<0.001	4191.5

Patient V20Gy (cc)

Treatment B (columns) / Treatment A (rows)

	CP5	CP6	CP7	CP8	CP9	CP10	CP11	CP12	CP13	CP14	CP15	NCP5	NCP6	NCP7	NCP8	NCP9	NCP10	NCP11	NCP12	NCP13	NCP14	NCP15	VMAT	B-VMAT
CP5	2050.	107.9	158.5	192.7	232.2	267.5	291.5	306.8	327.9	347.6	369.8	9.7	115.8	166.1	215.2	245.8	277.5	311.0	339.9	373.6	399.5	425.9	385.4	-60.7
CP6	<0.001	1943.	50.6	84.8	124.3	159.6	183.6	199.0	220.0	239.7	261.9	-98.2	7.9	58.2	107.4	137.9	169.6	203.1	232.0	265.7	291.6	318.0	277.5	168.6
CP7	<0.001	0.002	1892.	34.2	73.6	109.0	133.0	148.3	169.4	189.0	211.3	148.8	-42.8	7.5	56.7	87.3	119.0	152.4	181.4	215.1	241.0	267.3	226.8	219.3
CP8	<0.001	<0.001	<0.001	1858.	39.5	74.8	98.8	114.2	135.2	154.9	177.1	183.0	-76.9	-26.6	22.6	53.1	84.8	118.3	147.2	180.9	206.8	233.2	192.7	-253.4
CP9	<0.001	<0.001	<0.001	<0.001	1818.	35.4	59.4	74.7	95.7	115.4	137.7	222.4	116.4	-66.1	-16.9	13.6	45.3	78.8	107.8	141.4	167.4	193.7	153.2	-292.9
CP10	<0.001	<0.001	<0.001	<0.001	0.017	1783.	24.0	39.3	60.4	80.0	102.3	257.8	151.8	101.5	-52.3	-21.7	10.0	43.4	72.4	106.1	132.0	158.3	117.8	328.3
CP11	<0.001	<0.001	<0.001	<0.001	0.002	0.003	1759.	15.3	36.4	56.0	78.3	-281.8	175.8	125.5	-76.3	-45.7	-14.0	19.4	48.4	82.1	108.0	134.3	93.8	-352.3
CP12	<0.001	<0.001	<0.001	<0.001	<0.001	<0.001	0.005	1744.	21.1	40.7	63.0	-297.3	191.3	140.8	-91.6	-61.1	-29.4	4.1	33.1	66.7	92.7	119.0	78.5	-367.6
CP13	<0.001	<0.001	<0.001	<0.001	<0.001	<0.001	<0.001	<0.001	1723.	19.7	41.9	-318.2	212.3	161.8	112.7	-82.1	-50.4	-16.9	12.0	45.7	71.6	98.0	57.5	-388.6
CP14	<0.001	<0.001	<0.001	<0.001	<0.001	<0.001	<0.001	<0.001	<0.001	1703.	22.3	-337.8	231.8	181.5	132.3	101.8	-70.1	-36.6	-7.6	26.0	52.0	78.3	37.8	-408.3
CP15	<0.001	<0.001	<0.001	<0.001	<0.001	<0.001	<0.001	<0.001	<0.001	<0.001	1681.	360.3	254.3	203.8	154.6	124.0	-92.3	-58.9	-29.9	3.8	29.7	56.0	15.5	-430.6
NCP5	*	<0.001	<0.001	<0.001	<0.001	<0.001	<0.001	<0.001	<0.001	<0.001	<0.001	2041.	106.0	156.3	205.5	236.1	267.7	301.2	330.2	363.9	389.8	416.1	375.6	-70.5
NCP6	<0.001	*	*	0.003	<0.001	<0.001	<0.001	<0.001	<0.001	<0.001	<0.001	<0.001	1935.	50.3	99.5	130.0	161.7	195.2	224.2	257.8	283.8	310.1	269.6	176.5
NCP7	<0.001	*	*	*	0.016	0.002	<0.001	<0.001	<0.001	<0.001	<0.001	<0.001	<0.001	1884.	49.2	79.7	111.4	144.9	173.9	207.5	233.5	259.8	219.3	226.8
NCP8	<0.001	<0.001	0.016	*	*	0.035	0.006	0.002	<0.001	<0.001	<0.001	<0.001	<0.001	<0.001	1835.	30.5	62.2	95.7	124.7	158.3	184.3	210.6	170.1	-276.0
NCP9	<0.001	<0.001	0.002	0.017	*	*	*	0.014	0.002	<0.001	<0.001	<0.001	<0.001	<0.001	0.001	1805.	31.7	65.2	94.1	127.8	153.7	180.1	139.6	306.5
NCP10	<0.001	<0.001	<0.001	0.003	*	*	*	*	*	*	0.007	<0.001	<0.001	<0.001	<0.001	<0.001	1773.	33.5	62.4	96.1	122.0	148.4	107.9	338.2
NCP11	<0.001	<0.001	<0.001	<0.001	0.007	*	*	*	*	*	*	<0.001	<0.001	<0.001	<0.001	<0.001	<0.001	1740.	28.9	62.6	88.6	114.9	74.4	-371.7
NCP12	<0.001	<0.001	<0.001	<0.001	<0.001	0.011	*	*	*	*	*	<0.001	<0.001	<0.001	<0.001	<0.001	<0.001	<0.001	1711.	33.7	59.6	86.0	45.4	-400.6
NCP13	<0.001	<0.001	<0.001	<0.001	<0.001	0.002	0.008	0.015	*	*	*	<0.001	<0.001	<0.001	<0.001	<0.001	<0.001	<0.001	<0.001	1677.	25.9	52.3	11.8	-434.3
NCP14	<0.001	<0.001	<0.001	<0.001	<0.001	<0.001	<0.001	0.002	0.014	0.048	*	<0.001	<0.001	<0.001	<0.001	<0.001	<0.001	<0.001	<0.001	<0.001	1651.	26.3	-14.2	-460.3
NCP15	<0.001	<0.001	<0.001	<0.001	<0.001	<0.001	<0.001	<0.001	<0.001	0.003	0.019	<0.001	<0.001	<0.001	<0.001	<0.001	<0.001	<0.001	<0.001	<0.001	<0.001	1625.	-40.5	-486.6
VMAT	<0.001	<0.001	<0.001	<0.001	<0.001	<0.001	<0.001	<0.001	0.002	0.005	0.028	<0.001	<0.001	<0.001	<0.001	<0.001	0.002	0.035	*	*	*	0.042	1665.	446.1
B-VMAT	*	<0.001	<0.001	<0.001	<0.001	<0.001	<0.001	<0.001	<0.001	<0.001	<0.001	*	0.001	<0.001	<0.001	<0.001	<0.001	<0.001	<0.001	<0.001	<0.001	<0.001	<0.001	2111.7

[1] Breedveld S, Storchi P, Voet P, and Heijmen B. iCycle: Integrated, multicriterial beam angle, and profile optimization for generation of coplanar and noncoplanar IMRT plans. Med. Phys., 2012;39:951–963.

[2] Voet P, Dirkx M, Breedveld S, Fransen D, Levendag P, and Heijmen B. Towards fully automated multi-criterial plan generation: a prospective clinical study. Int. J. Radiat.Oncol. Biol. Phys., 2013;85:866–872.

[3] Breedveld S, Storchi P, and Heijmen B. The equivalence of multi-criteria methods for radiotherapy plan optimization. Phys. Med. Biol., 2009;54:7199–7209.

[4] Hussein M, Heijmen B. J. M, Verellen D, and Nisbet A. Automation in intensity modulated radiotherapy treatment planning-a review of recent innovations. Br J Radiol, 2018;91:20180270.

[5] Voet P, Dirkx M, Breedveld S, Al-Mamgani A, Incrocci L, and Heijmen B. Fully automated VMAT plan generation for prostate cancer patients. Int. J. Radiat. Oncol. Biol. Phys., 2014;88:1175–1179.

[6] Sharfo A. W, Dirkx M. L, Breedveld S, Méndez Romero A, and Heijmen B. J. VMAT plus a few computer-optimized non-coplanar IMRT beams (VMAT+) tested for liver SBRT. Radiother Oncol, 2017;123:49–56.

[7] Rossi L, Sharfo A. W, Aluwini S, Dirkx M, Breedveld S, and Heijmen B. First fully automated planning solution for robotic radiosurgery - comparison with automatically planned volumetric arc therapy for prostate cancer. Acta Oncol, 2018;57:1490–1498.

[8] Heijmen B, Voet P, Fransen D, et al. Fully automated, multi-criterial planning for Volumetric Modulated Arc Therapy - An international multi-center validation for prostate cancer. Radiother Oncol, 2018;128:343–348.

[9] Alber M and Reemtsen R. Intensity modulated radiotherapy treatment planning by use of a barrier-penalty multiplier method. Optim. Methods Softw., 2007;22:391–411.
