# Supplementary material for: On the Importance of Individualized, Non-Coplanar Beam Configurations in Mediastinal Lymphoma Radiotherapy, Optimized With Automated Planning
Source: Front Oncol. 2021 Apr 15;11:619929. doi: 10.3389/fonc.2021.619929 (PMC8082440; doi:10.3389/fonc.2021.619929)
Supplement: Supplementary file 3 [file Image_3.pdf]

Electronic supplement C

Inter-patient beam selection variations

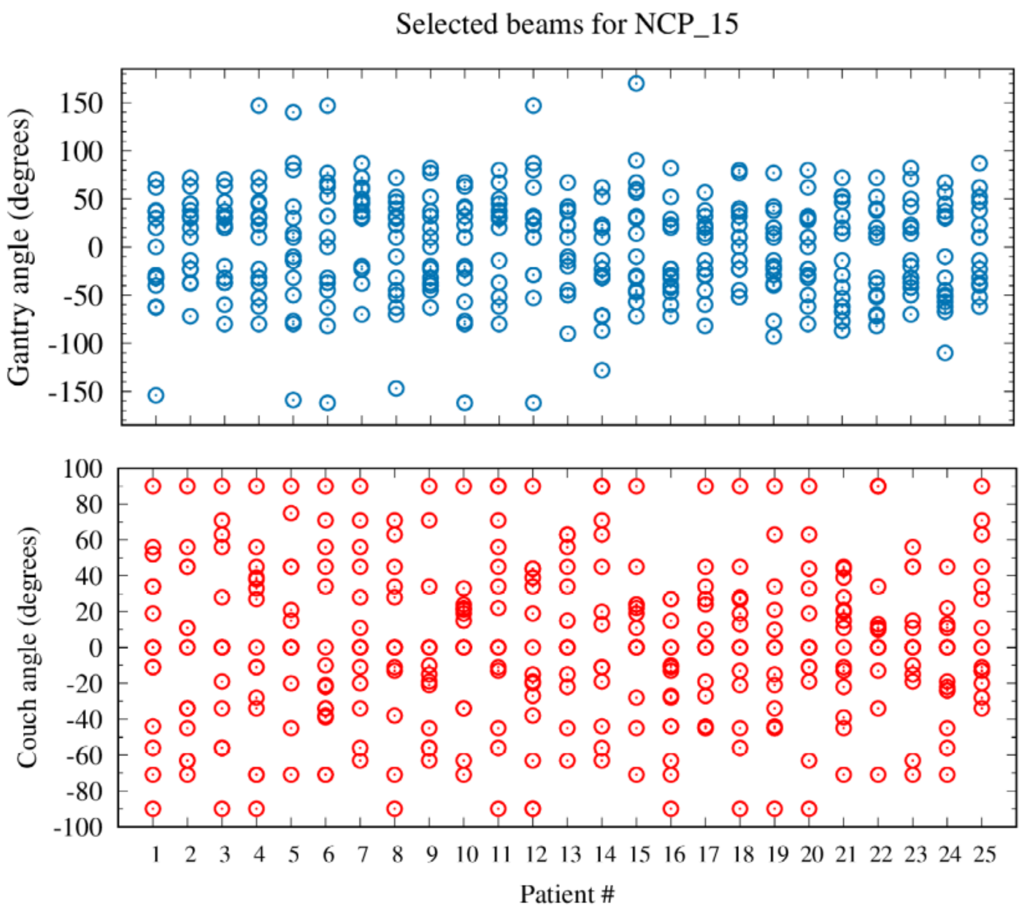

Figure C1. Optimized, patient-specific beam directions for NCP_15 plans for patients 1-25.

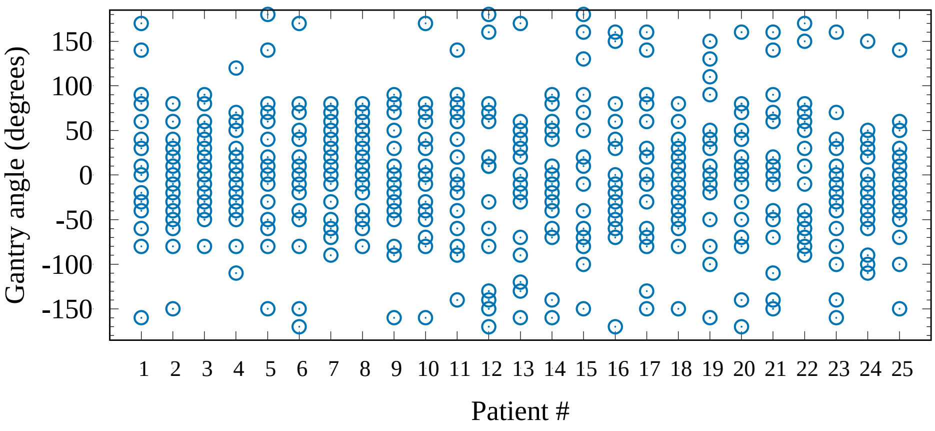

Figure C2. Optimized, patient-specific beam directions for CP_15 plans for patient 1-25.
